# Comprehensive analysis of non-synonymous single-nucleotide polymorphism of human *TSC1* and *TSC2* genes: An *in silico* approach

**DOI:** 10.1371/journal.pone.0343189

**Published:** 2026-09-03

**Authors:** Tasnim Alam, Shangida Akther

**Affiliations:** Department of Biochemistry and Molecular Biology, Shahjalal University of Science and Technology, Sylhet, Bangladesh; National University of Singapore, SINGAPORE

## Abstract

Tuberous sclerosis complex (TSC) is an autosomal dominant disorder resulting from the mutations in the *TSC1* and *TSC2* genes and is distinguished by benign hamartoma formation in multiple organs. The *TSC1*–*TSC2* complex regulates mTORC1 signaling in response to cellular growth conditions. This study aims to predict the structural stability and functional effects of non-synonymous single-nucleotide polymorphisms (nsSNPs) in human *TSC1* and *TSC2* using computational approaches. Twelve computational tools were assessed using receiver operating characteristic (ROC) analysis and applied to identify deleterious nsSNPs. Protein stability was predicted using I-Mutant 2.0 and MUpro, while evolutionary conservation was analyzed with ConSurf. NetPhos 3.1 identified potential PTM sites, and MutPred2.0 evaluated their functional impact. Project HOPE assessed mutation-induced physicochemical changes. Structural models were validated using multiple tools, visualized in ChimeraX 1.9, and further evaluated by molecular dynamics simulation to confirm wild-type and mutant stability. A combined in silico analysis identified twelve high-risk nsSNPs in *TSC1* and sixteen in *TSC2*, all reducing protein stability, located in conserved regions, and potentially disrupting phosphorylation sites. MutPred and Project HOPE confirmed their impact on protein function. Functional analysis showed *TSC1* and *TSC2* affect mTORC1 and PI3K–Akt pathways. RMSF and RMSD analyses revealed that *TSC1* variants rs1846545280 (G236E), and rs2132135678 (V234E), and *TSC2* variants rs45517223 (S758C), rs2151354925 (T836P), and rs45517365 (R1570W) had the largest structural fluctuations. Substitution with glutamic acid, a negatively charged and bulkier residue, may disrupt local folding of *TSC1*. Similarly, replacement of arginine with tyrosine at position 1570 may impair Rheb binding at the GAP domain of *TSC2*. These findings highlight potentially pathogenic nsSNPs in *TSC1* and *TSC2*.

## 1. Introduction

Tuberous Sclerosis Complex (TSC) is a multisystemic disorder and autosomal dominant tumor syndrome categorized by the development of various benign tumors and it most commonly affects the brain, kidneys, skin, heart, and lungs [1]. Functionally, the *TSC1/TSC2* complex is a crucial integrator of signals regulating protein translation and cell development, and it resides at the core of several growth signaling pathways [2]. The hamartin (encoded by TSC1), tuberin (product of the TSC2 gene), and the newly discovered TBC1D7, that creates significant oligomeric assemblies in cells, make up the TSC complex [3]. Structurally, hamartin (*TSC1*) and tuberin (*TSC2*) exhibit distinct functional domains that underpin their regulatory roles in cell growth and mTORC1 signaling. *TSC1* consists of an N‑terminal α‑helical/HEAT repeat region, a putative transmembrane segment, and extensive coiled‑coil regions in its central and C‑terminal portions that mediate heterodimerization with *TSC2* and complex assembly with TBC1D7 [4]. Additionally, N‑terminal region of *TSC1* has been found to do Rho GTPase regulation that contributes to cytoskeletal dynamics and focal adhesion [5]. *TSC2* is characterized by an extended HEAT repeat/α‑solenoid domain at the N‑terminus that interacts with *TSC1*, followed by a dimerization domain (DD), and a GTPase‑activating protein (GAP) domain [3].

Even though *TSC2*'s GAP activity is clearly important to the complex's function, *TSC1* is needed to keep *TSC2* stable and stop ubiquitin from breaking it down. [6]. *TSC2* has also been shown to physically interact with the transcription factor FoxO1 via a C‑terminal region linking *TSC2* to insulin signaling pathways and functional modulation of the *TSC1*‑*TSC2* complex [7]. Apart from variants affecting the *TSC2* GAP domain, missense mutations in *TSC1* or *TSC2* frequently disrupt the complex, resulting in *TSC2* degradation [8]. There are various types of SNPs, and the most destructive type is a missense SNP, which is a type of non-synonymous SNP (nsSNP) substitution defined by amino acid replacement with the likelihood of producing a mutant protein with novel structural and functional properties [9]. Such types of SNP may promote harmful activities by reducing protein solubility, disrupting protein tertiary structure, and changing gene regulation by affecting transcription regulatory proteins, causing cancer [10,11].

Over 90% of human nucleic acid sequence differences are caused by single-nucleotide polymorphisms (SNPs) [12]. The total amount of missense SNPs for every gene displayed a difference in the attribution to disease modification, despite the fact that many missense SNPs for multiple genes have been reported and deposited in databases. As a result, it became crucial to separate the SNPs with potential pathogenic activity from the group of neutral variants [13].

Numerous large cohorts of TSC patients have undergone thorough screening for mutations at each of the *TSC1* and *TSC2* loci, and numerous harmful mutations have been found. While missense mutations account for about 20% of the mutations found in the *TSC2* gene, they are far less common in the *TSC1* gene. To characterize pathogenic variants, mutational and molecular analysis had been performed in several different demographics such as Mexican [14], Brazilian [15], Chinese [16], Danish [17], Japanese [18], and Greek [19]. Similar to the pathogenic p.R611Q mutation, the missense variants drastically decreased *TSC1* and *TSC2* signals and failed to inactivate TORC1-dependent S6K-T389 phosphorylation. [20]. Perhaps as a result of conformational alterations or enhanced targeting of *TSC2* for destruction, three *TSC2* mutants namely R505Q, H597R, and L1624P demonstrated decreased affinity in binding to *TSC1* [21]. Missense mutations of TSC2 were also discovered to significantly reduce the TSC complex's ability to suppress S6K-T389 phosphorylation and p.S1045F and p.I1197F alterations disrupt the TSC complex, which boosts mTOR activity [22]. Several *TSC1* variants have been associated with different cancers. *TSC1* rs7874234 is hypothesized to be functional in ER+ breast cancer because the T allele, but not the C allele, may create an estrogen receptor element (ERE) site, resulting in increased *TSC1* transcription and subsequent inhibition of mTOR [23]. Missense variants present in the N-terminal domain of *TSC1*, were found to be linked with bladder cancer [2]. The *TSC1* rs13295634 (TG/GG) allele has been associated with the worst survival in colorectal cancer [24]. A missense variant p.Ser208Gly of *TSC1* is found to have a damaging impact in keratoconus patients [25].

The *TSC1* gene is less studied than *TSC2* as most disease-causing mutations are found in *TSC2* though *TSC1* is equally important in the formation of the TSC complex. The nsSNPs are unique among SNPs since they involve a single amino acid substitution inside a gene's coding region, which may result in notable changes to the protein's structural and functional properties [26]. In this context, computational approaches provide a cost-effective and time-efficient alternative to preliminary experimental screening, enabling the prioritization of functionally significant SNPs that may disrupt TSC complex stability or Rheb binding for subsequent experimental validation. This study aims to characterize functionally significant SNPs using *in silico* approaches*TSC1TSC2*as an initial step toward identifying the most deleterious variants in *TSC1* and *TSC2* across the human genome.

## 2. Methodology

nsSNPs data was retrieved from the NCBI-dbSNP database. To predict the most deleterious SNP, multiple computational tools of varying principles were employed. Finally, the predicted SNPs were analyzed for their role in protein stability.

### 2.1. Retrieving nsSNP data of *TSC1* and *TSC2* gene

The nsSNPs of the *TSC1* and *TSC2* genes were obtained from the National Center for Biotechnology Information's dbSNP database (https://www.ncbi.nlm.nih.gov/snp/). The UniProtKB database (https://www.uniprot.org/) provided the sequences of proteins Q92574 and P49815. Only missense variants of nsSNPs were selected during dataset preparation because, in contrast to nonsense variations, their effects can range from benign to extremely harmful, depending on the significance of the substituted amino acid in the structure or function of the protein. Because of their known capacity to profoundly affect protein structure and function, the coding regions of 2583 (*TSC1*) and 4468 (*TSC2*) nsSNPs were given priority for additional research. All of the nsSNPs were then separated and collected for additional research.

### 2.2. Validating deleterious nsSNP prediction tools

Data from pathogenic and benign variants of TSC*1* and TSC*2 were used* as experimental data to assess how the nsSNP Prediction tools differentiates between deleterious nsSNPs and normal nsSNPs. ClinVar data was used as a control to calculate AUC values as this database is the gold standard for containing annotated pathogenic mutations. Several prediction tools used in this study, including ClinPred, REVEL, BayesDel, and AlphaMissense. They were trained on variants from ClinVar. Variants overlapped with the training datasets of the evaluated predictors were not systematically excluded from the benchmark dataset. The benchmark dataset consisted of 1,416 ClinVar-annotated variants, including 1,271 pathogenic and 145 benign variants S1 Table in S1 File). ClinVar was accessed on 1^st^ january,2026. No filtering and deduplication was not applied as several rsIDs had multiple mutations with different amino acids. So variant-level overlap between the training datasets of these tools and the evaluation dataset could not be completely excluded; a degree of circular validation may be present. The pROC program in R was used to create receiver operating characteristic (ROC) curves. To evaluate each tool's prediction performance, the area under the curve (AUC) was computed [27]. Further pRROC package was used to calculate and visualize the precision-recall characteristics of the prediction tools [28]

### 2.3. Predicting deleterious variants

The ACMG guidelines and requirements suggest using various prediction software packages for sequence variant analysis since each in silico tool has its own set of strengths and drawbacks. To comprehensively evaluate the functional consequences of nsSNPs, a multi-tool approach containing diverse categories with consensus strategy, such as sequence homology-based model (Sift, Provean), sequence structure-based model (Fathmm, Polyphen-2), Supervised learning model (AlphaMissense, ClinPred, Vest4, M-cap), Meta predictor (CADD, BayesDel, MetaLR, REVEL) was employed. A total of twelve tools*TSC1TSC2* including PROVEAN (Protein Variation Effect Analyzer), SIFT (Sorting Intolerant From Tolerant), PolyPhen-2 (Polymorphism Phenotyping v2), FATHMM (Functional Analysis Through Hidden Markov Models), AlphaMissense, Bayesdel (Bayesian Deleteriousness), MetaLR (Meta-Logistic Regression), CADD, REVEL, VEST4, ClinPred, M-CAP were utilized. The cut-off score of PROVEAN is 2.5 [29] andSIFT is ≤ 0.05 [30]. A score exceeding those values was regarded as benign. The score for PolyPhen2 ranges from 0 to 1, with 0.45 to 0.95 being regarded as potentially harmful and 0.95 to 1.0 as likely harmful. [31]. Variants with a FATHMM score below −1.5 are classified as deleterious [32]. A score above a certain threshold (e.g., 0.56) suggests the variant is likely pathogenic, while a score below suggests it is likely benign in AlphaMissense [33]. The Bayesdel cut-off value varies from −1.29334 to 0.75731, with higher scores indicating more harmful variants [34]. ClinPred's decision boundary is 0.5, with lower scores indicating benignity and larger scores indicating pathogenicity [35]. MetaLR considers nsSNP as benign with scores of 0.5 or lower and vice versa [32]. CADD has “raw score” and a scaled PHRED-like score that ranks the variant relative to all potential replacements in the human genome. A higher score (>20) means a higher chance of deleteriousness [36]. The cutoff score for REVEL is > 0.5 [37]. The more detrimental the variant, the higher the VEST final score, which goes from 0 to 1 [32]. For M-cap, a variant is more harmful if its score is more than 0.025 [38]. This integrated method offered a solid framework for spotting possibly harmful variations. nsSNPs that were identified by all twelve tools as “Disease/Deleterious” were selected for additional examination.

### 2.4. Stability prediction of nsSNPs on protein

As the most deleterious nsSNPs alter protein structure and disrupt molecular interactions, the initially characterized deleterious nsSNPs undergo protein stability analysis. The possible impacts of nsSNPs on the protein's structural reliability and free energy change Delta Delta G (DDG) were estimated using I-Mutant2.0 and the MUpro web server. Stability will therefore rise when DDG is more than 0 kcal/mol and fall when DDG is less than 0 kcal/mol. In this project, protein sequence interface of I-Mutant2.0 was used. Protein sequence, AA position, Residue change was given as input. Output is displayed as either free energy change or reliability index at pH 7.0 and temperature 25^0^ indicating ideal environment with the statement increasing or decreasing stability of protein [39]. Both Support Vector Machines and Neural Networks are used in MUpro. After analysis, MUpro provides a confidence score from −1 to +1 anda negative score indicates a predicted decrease in protein stability and a positive score suggests increased stability. Results from both SVM and Neural networks were incorporated into the project [40]. Both types of results in the case of decreasing stability were integrated in this study.

### 2.5. Evolutionary conservation of deleteriousnsSNPs

Conservation surface map (ConSurf) is a computational tool designed for mapping evolutionarily conserved residues onto the surface of protein structures, providing insight into functionally or structurally important sites. It utilizes multiple sequence alignments (MSA). Residues essential for maintaining the protein's fold and function tend to be highly conserved, while variable regions tolerate substitutions more freely. On a scale of 1–9, the degree of conservation for each residue of the relevant protein is evaluated and classified as 1) variable (1–4), 2) intermediate (5–6), and 3) conserved (7–9). These mapped results help identify critical residues involved in structural stability, active sites, ligand binding, or protein-protein interaction interfaces, offering valuable guidance for functional annotation and mutational analysis [41]. As nsSNPs occurring at conserved regions are more likely to be disease-causing, nsSNPs in such regions were prioritized for downstream analysis.

### 2.6. Prediction of post-translational modification (PTM) sites

High-risk nsSNPs located within conserved regions and close to predicted PTM sites are more likely to interfere with normal post-translational modifications, thereby affecting protein stability and function, indicating higher pathogenic potential. NetPhos 3.1 is a computational tool used to predict potential phosphorylation sites on protein sequences, identifying serine (S), threonine (T), and tyrosine (Y) residues that may undergo phosphorylation employing an artif icial neural network (ANN)–based algorithm. The tool assigns a phosphorylation probability score (0–1) to each site, and a greater score (>0.5) indicates a greater likelihood of modification [42].

### 2.7. Ientification of structural and functional alterations

While PTM analysis characterized the high-risk deleterious variants, MutPred2 subsequently utilized assessed their functional impacts at the molecular level. MutPred2 utilized a neural network–based algorithm from on a large dataset of 53,180 pathogenic variants and 206,946 neutral variants from HGMD, SwissVar, and dbSNP. The tool outputs a g score (ranging from 0 to 1), where values >0.5 indicate a higher probability of the variant being deleterious. Additionally, it provides empirical p-values for various potential molecular consequences (e.g., loss of phosphorylation, altered secondary structure), indicating whether the mutation is likely to cause significant changes in specific biological properties [43].

### 2.8. Structural impact of SNPs on proteins

Project HOPE was employed to further assess the potential structural alteration and biophysical changes in the protein caused by the high-risk deleterious nsSNPs based on the 3D structure. The HOPE server collects information from a variety of sources, in addition to constructing homology models with the YASARA program to execute the required function [44]. It explores how amino acid composition alterations affect native structures, as well as differences in hydrophobicity, charge, and size between wild-type and mutant residues. Uniprot provides Project HOPE with the *TSC1* and *TSC2* protein sequences to estimate the altered protein's 3D structure.

### 2.9. Prediction of destabilizing effects of nsSNPs in *TSC1* and *TSC2* gene

DUET was used to assess the structural stability effects of the high-risk deleterious nsSNPs on on their 3D structure. DUET combines two complementary computational approaches—mCSM (graph-based signatures) and SDM (amino acid environment-specific substitution tables) using Support Vector Machines (SVM) to provide a consensus prediction. It uses the 3D structure to analyze the residue's microenvironment. The result is given based on thermodynamic stability which is free energy change, Delta Delta G (DDG). Stability will therefore rise when DDG is more than 0 kcal/mol and fall when DDG is less than 0 kcal/mol. It helps to evaluate the effect of a single mutation on the stability of proteins and functions [45].

### 2.10. Kaplan–Meier survival analysis of *TSC1* and *TSC2* gene expression

The relationship between TSC1 and TSC2 gene expression levels and clinical survival outcomes was assessed using Kaplan-Meier survival analysis. The median value was used to categorize patients into high- and low-expression groups. Kaplan-Meier survival curves were used to measure overall survival (OS), and the log-rank test was used to determine statistical significance. Statistical significance was defined as a p-value of less than 0.05 [46].

### 2.11. Interaction of *TSC1* and *TSC2* with other genes and proteins

i) The enrichR tool was used to detect statistically significant (P < 0.05) correlations between the input gene set and curated illness databases (DisGenet) and complementing functional pathways (KEGG) [47]. RStudio (4.2.2) was employed for this analysis. The GGplot2 library of R was used for visualization.
ii) The STRING database) predicts the PPI network of the *TSC1* and *TSC2* protein (https://string-db.org/). Network was visualized using Cytoscape software. It provides us with insights into how nsSNPs in *TSC1* and *TSC2*-associated genes may affect underlying biological pathways and molecular functions, indicating their potential role in disease pathogenesis.

### 2.12. Comparative 3D modeling of *TSC1, TSC2* and its mutant

To illustrate the structural impact of nsSNP, comparative 3D modeling of *TSC1, TSC2*, and their mutant structures was carried out. This allowed for the visualization of the mutant structure's divergence from the wild type. Alphafold, Phyre2, and Swiss-Model were used to generate wild-type protein structures of *TSC1* and *TSC2*. The Alphafold *TSC1* structure was used as the wild type [48]. The fold recognition method was adapted to model the 3D structure of the Tuberin domain of *TSC2* due to the absence of a homologous structure. Phyre2 was utilized to produce the three-dimensional structure of the Tuberin domain [49]. The template, c4jZ7A, was used by Phyre2 for the estimation of three-dimensional models and then, the visualization of the structures was done by ChimeraX 1.6. Swiss-Model modeled the Gap domain as close homologs were available [50]. All models were evaluated with ERRAT, MolProbity, Verify 3D, and PROCHECK [51]. Mutant protein structures were generated using ChimeraX 1.6, a molecular visualization and modeling tool that allows interactive mutagenesis [52]. After substituting the wild-type amino acid with the desired mutant residue, the local conformation of the mutant side chain was optimized using the Dunbrack 2010 rotamer library. This step samples the most favorable side-chain conformations (rotamers) for the new residue based on statistically preferred dihedral angles from high-resolution crystal structures, minimizing steric clashes and unfavorable torsions. Once the mutant model was built and side-chain conformation optimized, mutant protein structure was superimposed onto the native (wild-type) protein structure using ChimeraX’s structural alignment tool. This superimposition allows for the measurement of the Root Mean Square Deviation (RMSD), which is a quantitative measure of structural similarity between two structures. A lower <0.2 score suggests a stable and realistic mutant model.. GalaxyWEB (https://galaxy.seoklab.org/) was used to minimize energy in each model in order to improve the structure, reduce steric conflicts, and ensure adequate stereochemical quality [53]. Specially for the proline substituted models, Rosetta Relax program of ROSIE was used to generate the model.[54].

### 2.13. Molecular dynamics simulation

Based on preliminary protein stability predictions, conservation analysis, PTM analysis, and destabilization analysis, nsSNPs in the Rhogtpase domain of *TSC1*, along with the nsSNPs in both Tuberin and Gap domains, were chosen for comprehensive molecular dynamics simulation (MDS). Molecular dynamics simulations were conducted for 100 ns to investigate the structural deviation of mutant proteins relative to the wild-type. GROMACS is a high-performance molecular dynamics (MD) simulation software used to model the physical movements of atoms and molecules over time, based on classical Newtonian mechanics [55]. Initially, each protein was placed within a simulation box solvated with explicit water molecules (TIP3P water models) and neutralized with appropriate counter-ions (Na⁺ and Cl−) to replicate physiological conditions. The system underwent energy minimization to eliminate unfavorable steric clashes and optimize atomic positions by achieving a local minimum in the potential energy landscape. Following this, two equilibration phases were conducted: NVT equilibration, maintaining a constant number of particles, volume, and temperature to stabilize the thermal environment, and NPT equilibration, keeping the number of particles, pressure, and temperature constant to stabilize system pressure and density while allowing solvent and ions to adjust around the protein structure. Finally, a 100-nanosecond production MD run was carried out, during which atomic forces were calculated and Newton’s equations of motion integrated at each time step (typically 1–2 femtoseconds)**.** Atomic positions, velocities, and energies were recorded at defined intervals, generating trajectory files for subsequent analysis of structural deviations, flexibility, and stability. Root Mean Square Deviation (RMSD) analysis was then used to monitor structural deviations from the initial conformation, with stable RMSD profiles indicating structural integrity. Further Root Mean Square Fluctuation (RMSF) analysis was used to directly compare the dynamic flexibility of native and mutant proteins under simulated physiological conditions.

## 3. Result

### 3.1. Retrieving missense variants of nsSNPs of *TSC1* and *TSC2* genes

**Fig 1:**
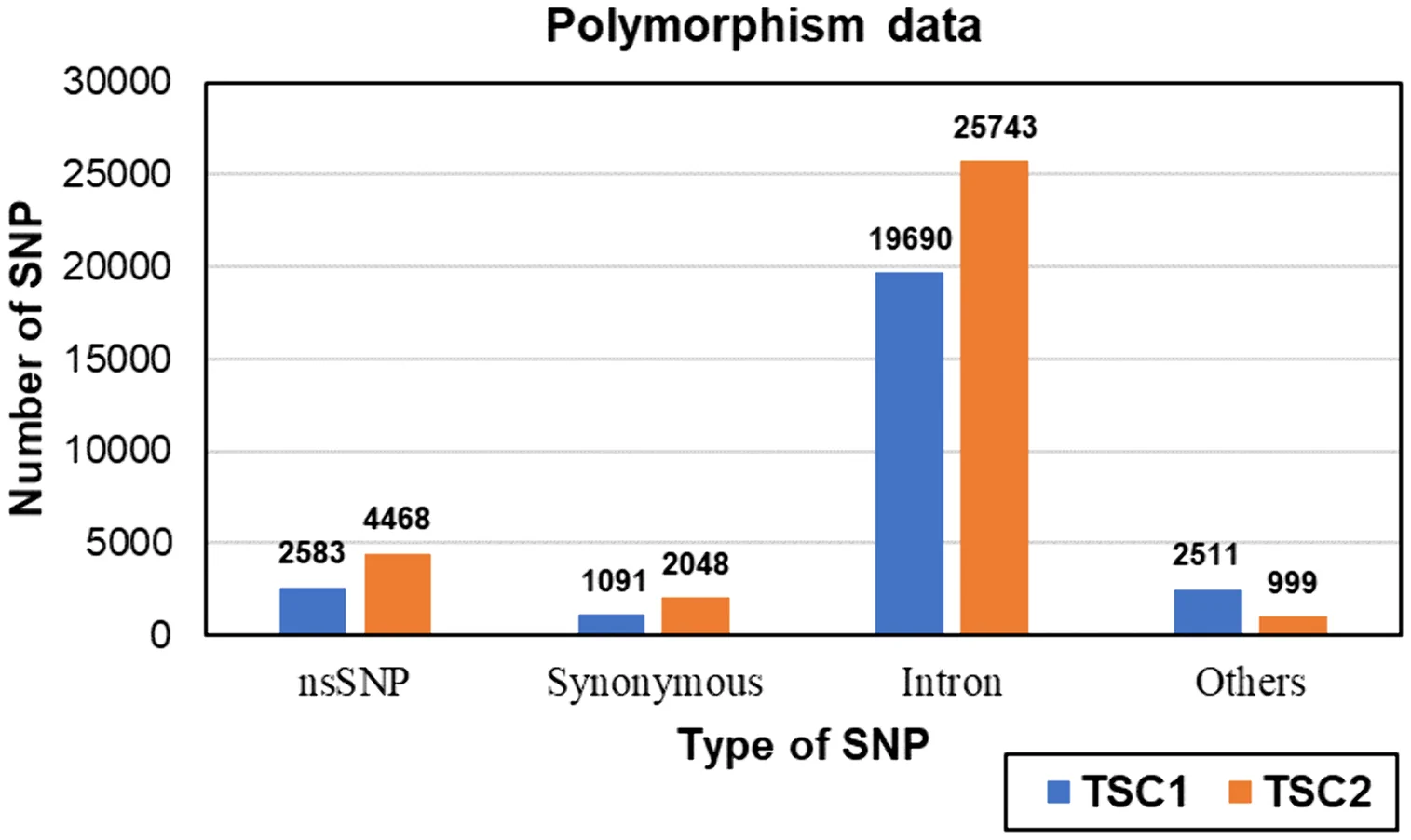
Piechart displaying each type of SNP in both *TSC1* and *TSC2.* In *TSC1*, there are 2583 non-synonymous SNP, 1091 synonymous SNP, 19,690 intron and 2511 other. 4468 non-synonymous SNP, 2048 synonymous, 25473 intron, and 999 others in *TSC2*.

The NCBI dbSNP database was employed to get polymorphism data for the *TSC1* and *TSC2* genes. There were 25,875 SNPs in *TSC1*, out of which 1,091 are synonymous variants, 19,690 intron region variants, 2583 missense variants of nsSNPs, and the remaining were different types. There were 33,258 SNPs in *TSC2*, containing 2,048 synonymous variants, 25,743 intron region variants, 4468 missense variants of nsSNPs, and the remaining were different types (Fig 1). All other SNPs were excluded except 2583 (*TSC1*) and 4468 (*TSC2*) non-synonymous SNPs (missense variations) for this project.

### 3.2. Assessing the specificity of *in-silico* pathogenicity prediction tools

**Fig 2:**
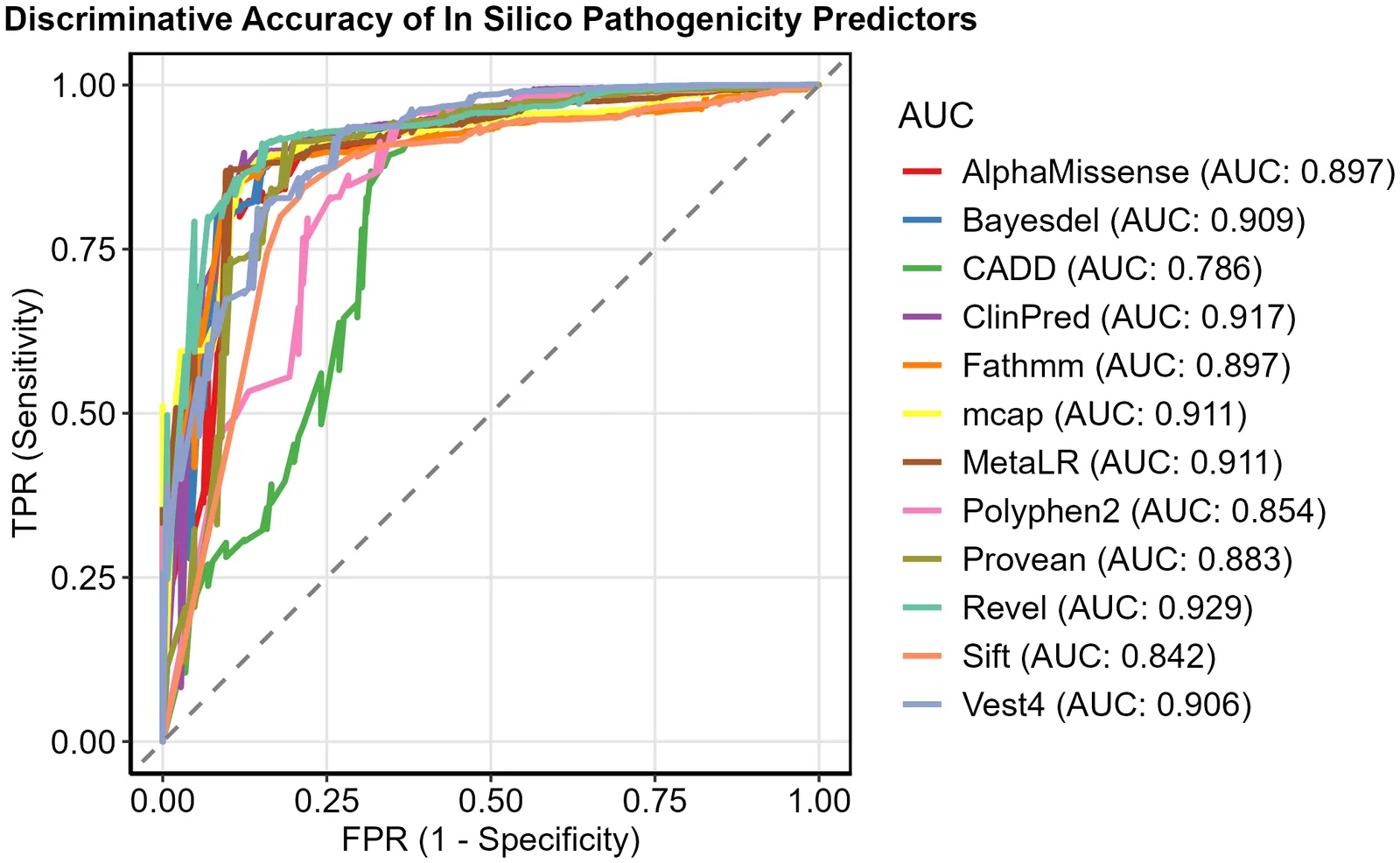
ROC curve displaying the accuracy of each nsSNP prediction model. TPR means True positive rate and FPR means False Positive Rate. FPR > 0.5 for every tools.

The performance of twelve computational tools in discriminating between pathogenic and benign variants was assessed using the Receiver Operating curve (ROC). The overall identification ability of each tool was quantified by the Area Under the Curve (AUC).. Tools with the highest AUC scores were ClinPred (AUC: 0.917), mcap (AUC: 0.911), and Revel (AUC: 0.926). These three tools exhibit highest specificity to rank pathogenic variants than benign variants compared to the other predictors such as Bayesdel (AUC: 0.909), AlphaMissense (AUC: 0.897), Sift (AUC: 0.842), CADD (AUC: 0.786), Fathmm (AUC: 0.897), Polyphen-2 (AUC:0.854), Vest4 (AUC: 0.9060), MetaLR (AUC: 0.911) and lastly Provean (AUC: 0.883) (Fig 2). Furthermore precision-recall curves demonstrated that REVEL achieved the highest AUPRC (0.986208117) followed by ClinPred (0.985016106) and AlphaMissense (0.984212205). BayesDel (AUPRC: 0.979715007), MetaLR (AUPRC: 0.983313485), mcap (AUPRC: 0.980690663), Vest4 (AUPRC: 0.982054892), Polyphen2 (AUPRC: 0.977932176) and CADD (AUPRC: 0.957832577) also showed strong predictive performance. Sift (AUPRC: 0.786399587), Fathmm (AUPRC: 0.776552793) and Provean (AUPRC: 0.766781031) showed comparatively lower AUPRC values, indicating comparatively reduced precision-recall performance. Overall, ensemble and machine learning-based predictors consistently higher performance within the evaluated dataset (S1 Fig).

### 3.3. Identification of deleterious nsSNPs

A comprehensive computational approach employing twelve diverse *in silico* algorithms which was usedto accurately identify deleterious non-synonymous single-nucleotide polymorphisms (nsSNPs). These algorithms, including sequence homology-based tools like SIFT (score ≤ 0.05) and PROVEAN (score ≤ −2.5), and those incorporating sequence and structural features such as PolyPhen-2 (score ≥ 0.85) and FATHMM (score < 0), were selected for their varied prediction mechanisms. Supervised machine learning models like AlphaMissense (score >0.5), VEST4 (score ≥ 0.5), M-CAP (score ≥ 0.025), and ClinPred (score ≥ 0.5) were also included, alongside ensemble meta predictors like MetaLR (score ≥ 0.5), BayesDel (score ≥ 0.2), REVEL (score ≥ 0.5), and CADD (PHRED score ≥ 20), which integrate multiple predictors for enhanced accuracy. Two datasets, comprising 2,583 nsSNPs in the *TSC1* gene and 4,468 nsSNPs in the *TSC2* gene, were analyzed. The integrated predictions from all twelve tools identified a total of 484 harmful nsSNPs in *TSC1* and 1,268 harmful nsSNPs in *TSC2*. After a comprehensive in-silico analysis to get the most damaging nSNPs, a final set of twelve nsSNPs was identified as the most potentially damaging variants in *TSC1* and sixteen in the *TSC2* gene. These include rs1846545280, rs1846672141, rs2131961079, rs2132135077, rs2132135600, rs2132235475, rs2131703748, rs2132003126, rs2132003339, rs2132135678, rs2132135839, and rs2132154057 in *TSC1* gene (Table 1).

**Table 1:** A compilation of 12 deleterious nsSNPs in the *TSC1* gene.

SNP	CADD	Alpha-Missense	BayesDel	Poly-phen2	ClinPred	SIFT	MetaLR	Provean	Fathmm	Revel	M-cap	Vest4
rs1846545280	33	0.9585	0.432	0.975	0.98	0.004	0.794	−3.05	−2.31	0.854	0.911	0.811
rs1846672141	28.4	0.9965	0.486	0.999	0.99	0	0.875	−6.52	−3	0.971	0.998	0.84
rs2131961079	32	0.7424	0.389	0.997	0.97	0.001	0.821	−2.97	−2.53	0.827	0.901	0.851
rs2132135077	31	0.8024	0.355	0.979	0.99	0.001	0.802	−4.02	−2.25	0.757	0.935	0.844
rs2132135600	31	0.7899	0.444	0.964	0.99	0.014	0.830	−4.49	−2.5	0.805	0.941	0.898
rs2132235475	31	0.9765	0.470	0.995	0.99	0.001	0.854	−5.1	−2.87	0.948	0.965	0.891
rs2131703748	29.4	0.6553	0.411	0.999	0.96	0.015	0.728	−3.3	−1.96	0.832	0.985	0.871
rs2132003126	28.8	0.9725	0.526	0.999	0.99	0.001	0.914	−4.98	−3.48	0.96	0.898	0.854
rs2132003339	32	0.9763	0.475	0.989	0.99	0.001	0.848	−4.56	−2.66	0.884	0.952	0.911
rs2132135678	29.2	0.9098	0.452	0.998	0.99	0.002	0.835	−4.92	−2.51	0.885	0.995	0.868
rs2132135839	32	0.9969	0.472	0.996	0.99	0	0.849	−5.38	−2.72	0.925	0.895	0.877
rs2132154057	32	0.9568	0.436	0.946	0.98	0.01	0.830	−3.48	−2.39	0.86	0.89	0.835

CADD > 20, Alpha missense > .5, BayesDel ≥ 0.2, PolyPhen-2 ≥ 0.85, ClinPred ≥ 0.5, SIFT ≤ 0.05, MetaLR ≥ 0.5, PROVEAN ≤ −2.5, FATHMM < 0, REVEL ≥ 0.5, M-CAP ≥ 0.025, and VEST4  ≥ 0.5 are threshold value to predict deleterious nsSNP

The nsSNPs in *TSC2* are rs45517223, rs45517365, rs45517381, rs372918437, rs397514980, rs397515030, rs397515159, rs397515313, rs761588986, rs1163066622, rs1389060581, rs1555438434, rs1555438551, rs1555510327, rs2151354925, and rs2151550678 (Table 2). These variants consistently surpassed the deleterious prediction thresholds across all applied tools and remained as high confidence damaging nsSNPs after the final integrative analysis.

**Table 2:** A compilation of sixteen deleterious nsSNPs in the *TSC2* gene.

SNP	CADD	Alpha-Missense	BayesDel	Poly-phen2	ClinPred	SIFT	MetaLR	Provean	Fathmm	Revel	M-cap	Vest4
rs45517223	28.6	0.595	0.400	0.996	0.956	0.002	0.765	−3.27	−2.24	0.75	0.811	0.911
rs45517365	24.2	0.969	0.435	0.977	0.986	0.005	0.853	−5.92	−3.63	0.685	0.84	0.998
rs45517381	22.9	0.996	0.195	0.998	0.996	0.001	0.928	−5.46	−3.91	0.684	0.851	0.901
rs372918437	24.6	0.667	0.353	0.993	0.995	0	0.821	−4.08	−2.97	0.732	0.844	0.935
rs397514980	25.1	0.987	0.476	0.985	0.989	0.003	0.878	−5.04	−3.6	0.891	0.898	0.941
rs397515030	34	0.994	0.494	0.977	0.992	0.001	0.889	−4.58	−3.53	0.814	0.891	0.965
rs397515159	25.2	0.979	0.489	0.995	0.994	0	0.874	−4.55	−3.41	0.874	0.871	0.985
rs397515313	32	0.967	0.539	0.974	0.993	0	0.925	−5.15	−3.97	0.944	0.854	0.898
rs761588986	33	0.908	0.428	0.992	0.916	0.006	0.777	−2.81	−2.29	0.767	0.911	0.952
rs1163066622	26.4	0.8564	0.436	0.982	0.974	0.006	0.815	−3.64	−2.6	0.664	0.868	0.995
rs1389060581	23.7	0.9866	0.425	0.951	0.994	0.004	0.829	−5.01	−3.56	0.742	0.877	0.895
rs1555438434	32	0.5831	0.319	0.996	0.984	0.003	0.837	−4.06	−3.3	0.775	0.835	0.89
rs1555438551	35	0.8824	0.537	0.999	0.999	0.004	0.948	−4.89	−4.21	0.951	0.821	0.851
rs1555510327	32	0.9208	0.461	0.992	0.994	0.001	0.823	−4.19	−2.73	0.697	0.855	0.899
rs2151354925	26.2	0.9258	0.487	0.996	0.997	0.001	0.840	−4.98	−2.87	0.915	0.921	0.872
rs2151550678	29.3	0.7511	0.382	0.958	0.971	0.005	0.793	−2.6	−3.32	0.776	0.875	0.891

CADD > 20, Alpha missense > .5, BayesDel ≥ 0.2, PolyPhen-2 ≥ 0.85, ClinPred ≥ 0.5, SIFT ≤ 0.05, MetaLR ≥ 0.5, PROVEAN ≤ −2.5, FATHMM < 0, REVEL ≥ 0.5, M-CAP ≥ 0.025, and VEST4  ≥ 0.5 are threshold value to predict deleterious nsSNP

### 3.4. Verification of high-risk deleterious nsSNPs by determining protein stability

484 and 1268 nsSNPs previously identified as harmful in *TSC1* and *TSC2*, respectively and were evaluated using two additional tools: I-Mutant 2.0 and MUpro to assess their deleterious impact on protein structure such as altered protein structure indicated by change in free energy. Based on the reliability index (RI) and expected changes in free energy (ΔΔG), I-Mutant analysis revealed that 350 and 750 nsSNPs reduced protein stability in TSC1 and TSC2, respectively. The change in Gibbs free energy following mutation is represented by the ΔΔG values (kcal/mol). Negative values (DDG < 0) suggest decreased protein stability, while positive values (DDG > 0) suggest increased stability. MUpro predicted that 300 nsSNPs destabilized the protein in *TSC1*, while 710 nsSNPs destabilized *TSC2*. For further analysis, missense variants of nsSNPs predicted to cause destabilization by both tools were selected. nsSNP selected by both tools are 250 in *TSC1* and 650 in *TSC2*. In TSC1, S201T and T235S show the highest negative DDG value of −2.31 and S1207N of TSC2 displays −2.13 in the I-mutant2.0 tool, indicating stronger destabilizing effects than other variants with lower negative DDG values. T1203I and T1023L of TSC2 had −2.31 and −2.15 DDG value respectively. Finally selected twelve nsSNPs of *TSC1* and sixteen nsSNP of *TSC2* are presented in Table 3 and Table 4.

**Table 3:** Impact of nsSNP on *TSC1* protein stability.

SNP	A.A. position	I-mutant2.0			MUpro	
		Prediction	Reliability index	DDG Value(kcal/mol)	Prediction	DDG Value(kcal/mol)
rs1846545280	G236E	Decrease	7	−1.19	Decrease	−0.892
	G236A		8	−1.2		−0.133
rs1846672141	L161P	Decrease	7	−1.4	Decrease	−1.51
rs2131850671	I482K	Decrease	7	−0.7	Decrease	−1.18
	I482R		8	−0.8		−1.49
rs2131961079	S361C	Decrease	6	−0.99	Decrease	−1
	S361F		7	−0.91		−1
rs2132135077	S237Y	Decrease	7	−0.53	Decrease	−1.32
	S237C		6	−0.91		−1.27
	S237F		6	−0.67		−1.51
rs2132135600	T235S	Decrease	8	−2.31	Decrease	−1.19
	T235I		9	−1.9		−1.45
rs2132235475	L90P	Decrease	7	−1.2	Decrease	−1.21
rs2131703748	L828H	Decrease	8	−1.15	Decrease	−1.2
rs2132003126	L264Q	Decrease	8	−1	Decrease	−0.95
rs2132003339	S263F	Increase	3	1.1	Decrease	−0.81
	S263C	Decrease	8	−1.5		−0.62
rs2132135678	V234E	Decrease	7	−0.98	Decrease	−1.15
rs2132154057	S201P	Increase	4	1	Increase	0.7
	S201T	Decrease	7	−2.31	Decrease	−1.5

DDG values (kcal/mol) represent the change in Gibbs free energy upon mutation. Negative values (DDG < 0) suggest decreased protein stability, while positive values (DDG > 0) suggest increased stability

**Table 4:** Impact of nsSNP on *TSC2* protein stability.

SNP	A.A. Change	I-mutant 2.0			MUpro	
		Prediction	Reliability Index	DDG Value(kcal/mol)	Prediction	DDG Value(kcal/mol)
rs45517223	S758C	Decrease	6	−1	Decrease	−0.9
	S758F	Increase	5	0.75	Increase	0.85
rs45517365	R1570G	Decrease	7	−1.2	Decrease	−1
	R1570W		8	−1.35		−1.25
rs45517381	S1653F	Decrease	7	−0.95	Decrease	−1.11
	S1653C		6	−1.4		−1.35
rs372918437	T1804P	Decrease	7	−1.5	Decrease	−0.95
rs397514980	R1570T	Decrease	7	−1.2	Decrease	−2
	R1570L		8	−1.4		−1.7
	R1570M		7	−1.6		−1.9
rs397515030	T1203K	Decrease	5	−0.8	Decrease	−1
	T1203I		7	−1.5		−2.31
	T1203R		8	−1.25		−1.5
rs397515159	S1653P	Increase	5	1.3	Increase	0.75
	S1653T	Decrease	6	−1.6	Decrease	−1.5
rs397515313	S1207I	Increase	6	1.5	Increase	1
	S1207N	Decrease	7	−2.13	Decrease	−2
rs761588986	A835G	Decrease	7	−1	Decrease	−1
	A835D		8	−0.5		−1.3
	A835V		9	−0.8		−1.6
rs1163066622	S1130C	Decrease	7	−0.9	Decrease	−1.1
	S1130F		7	−0.75		−0.95
rs1389060581	R1570S	Decrease	6	−1	Decrease	−0.9
rs1555438434	T1627N	Decrease	6	−0.68	Decrease	−0.75
	T1627S		8	−1.31		−1
	T1627I		5	−1.5		−0.95
rs1555438551	R1570C	Decrease	8	−1.2	Decrease	−0.67
	R1570G		8	−1.7		−1.3
rs1555510327	T1023R	Decrease	7	−1	Decrease	−0.58
	T1023L		7	−1.25		−2.15
	T1023M		8	−1.75		−1.45
rs2151354925	T836S	Decrease	8	−1.21	Decrease	−1.31
	T836P		8	−1.61		−1.25
	T836A		7	−1.55		−1.87
rs2151550678	S1507T	Decrease	7	−1.54	Decrease	−1.3
				
	S1507P		6	−1.29		−0.77
	S1507A		9	−1.67		−1

DDG values (kcal/mol) represent the change in Gibbs free energy upon mutation. Negative values (DDG < 0) suggest decreased protein stability, while positive values (DDG > 0) suggest increased stability

### 3.5. Evolutionary conserved deleterious nsSNPs of both *TSC1* and *TSC2* gene

A clear pattern of highly conserved residues was identified by sequence conservation analysis. Most of these residues were projected to be exposed in TSC2, while most were predicted to be buried in TSC1 (b). The distribution of functional residues (f), which are characterized as highly conserved and exposed, throughout the sequence suggests that they are involved in important functional locations. On the other hand, structural residues (s), which are buried and highly conserved, emphasize their crucial functions in preserving the stability of proteins. Highly conserved regions hold critical roles in maintaining the molecule’s function and structure, where changes are often deleterious, while more variable regions tolerate mutations without significantly compromising the macromolecule’s performance [56].ConSurf analysis revealed that 20 nsSNPs are located within highly conserved region and the rest in variable regions. Only SNPs in highly conserved regions were selected for further analysis as they are most likely to have a functional consequence on the protein structure. Among the final twelve nsSNPs of *TSC1*, G236, S361, T235, and S263 are considered functional, highly conserved and exposed residues. L161, I482, S237, L90, L828, L264, V234, and S201 are considered highly conserved, structural, and buried residues (Table 5).

**Table 5:** Conservation score of nsSNPs in *TSC1.*

SNP Id	Position	Residue	Consurf score	Feature	Buried/Exposed	Functional/Structural
rs1846545280	236	G	−0.664	9, highly conserved	e	f
rs1846672141	161	L	−0.667	9, highly conserved	b	s
rs2131961079	482	I	−0.72	9, highly conserved	b	s
rs2132135077	361	S	−0.728	9, highly conserved	e	f
rs2132135600	237	S	−0.728	9, highly conserved	b	s
rs2132235475	235	T	−0.723	9, highly conserved	e	f
rs2131703748	90	L	−0.667	9, highly conserved	b	s
rs2132003126	828	L	−0.668	9, highly conserved	b	s
rs2132003339	264	L	−0.667	9, highly conserved	b	s
rs2132135678	263	S	−0.728	9, highly conserved	e	f
rs2132135839	234	V	−0.718	9, highly conserved	b	s
rs2132154057	201	S	−0.728	9, highly conserved	b	s

ConSurf score > −4 means highly conserved region. b = buried residue, e = exposed residue, s = structural residue, f = functional residue. Exposed residues are also functional, and buried residues are also structural residues.In case of TSC2, ConSurf shows that 27 nsSNPs are in highly conserved regions. Among the final sixteen nsSNPs of TSC2, S758, R1570, S1653, T1804, S1203, T1627, T1023) are revealed as exposed, highly conserved and functional residues. S1207, A835, S1130, T836, and S1507 are buried, highly conserved, and structural (Table 6).

**Table 6:** Conservation score output of nsSNPs in *TSC2.*

SNP Id	Position	Residue	ConSurf score	Feature	Exposed/Buried	Structural/Functional
rs45517223	758	S	−0.512	9, highly conserved	e	f
rs45517365	1570	R	−0.492	9, highly conserved	e	f
rs45517381	1653	S	−0.512	9, highly conserved	e	f
rs372918437	1804	T	−0.508	9, highly conserved	e	f
rs397514980	1570	R	−0.492	9, highly conserved	e	f
rs397515030	1203	T	−0.508	9, highly conserved	e	f
rs397515159	1653	S	−0.512	9. highly conserved	e	f
rs397515313	1207	S	−0.512	9, highly conserved	b	s
rs761588986	835	A	−0.504	9, highly conserved	b	s
rs1163066622	1130	S	−0.512	9, highly conserved	b	s
rs1389060581	1570	R	−0.492	9, highly conserved	e	f
rs1555438434	1627	T	−0.508	9, highly conserved	e	f
rs1555438551	1570	R	−0.492	9, highly conserved	e	f
rs1555510327	1023	T	−0.508	9, highly conserved	e	f
rs2151354925	836	T	−0.508	9, highly conserved	b	s
rs2151550678	1507	S	−0.512	9, highly conserved	b	s

ConSurf score > −4 means highly conserved region. b = buried residue, e = exposed residue, s = structural residue, f = functional residue. Exposed residues are also functional, and buried residues are also structural residues.

### 3.6. Disruption of post-translational modification (PTM) sites by deleterious nsSNPs

**Fig 3:**
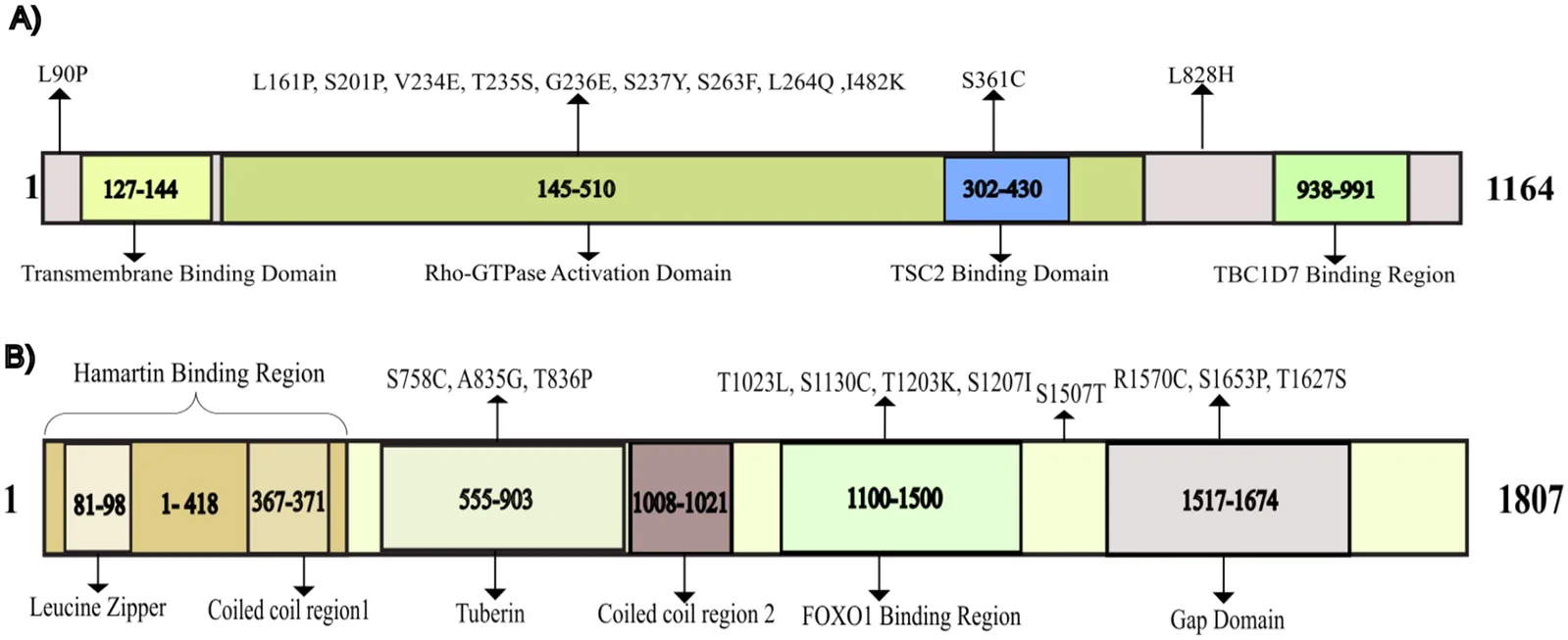
Predicted Phosphorylation site in *TSC1* and *TSC2.* A) S201P, T235S, S237Y, S263F, G236E, L161P, S237Y, L264Q, V234E, I482K are in Rho-GTPase activating domain. S361C is in the *TSC2* binding domain. L828H is in the NFL binding domain.B) S758C, A835G, and T836P are in the tuberin region. T1023L, S1130C, T1203K, S1207I, and S1507T are present on FOXO1 binding region. R1570C, T1627S, S1653P, and T1804P are in the GAP domain.

High-risk nsSNPs with attribute of being within conserved regions and close to predicted PTM sites are more likely to have higher pathogenic potential as they can disrupt the protein’s folding, thus affecting its 3D structure. NetPhos 3.1 was employed to get phosphorylation sites in both *TSC1* and *TSC2*. The threshold value for any amino acid position to be predicted as a phosphorylation site is > 0.5. Significant Phosphorylation sites of *TSC1* and *TSC2* are shown in Fig 3A and Fig 3B respectively. Result table is given in supporting information (S2 Table in S2 File).

### 3.7. Identification of functional and structural modifications

The 12 most damaging SNPs of *TSC1* and 16 of *TSC2* were submitted to the MutPred web server and the findings with probability scores are given in S3 Table and S4 Table in S2 File, respectively. All nsSNPs show strong pathogenic potential with g value above 0.6 and p-value less than 0.05. This analysis reveals these point mutation may affect proteins’ interaction, binding affinity and structural arrangement thus enhancing their pathogenic potential.

### 3.8. Structural Impact of High-Risk nsSNPs on Proteins

The HOPE server evaluates how a mutation impacts the structural variation of a protein in terms of size, charge, hydrophobicity, and spatial structure when compared to the wild type. All twelve nsSNPs altered amino acid size. Three mutant amino acids (G236E/A, I482K/R, V234E) exhibited both increased size and less hydrophobicity compared to their wild-type counterparts in *TSC1*. Five nsSNPs (S237C/Y/F, T235I/S, L828H, L264Q, S263C/F) were found to be both of increased size and more hydrophobic. Two (L161P, L90P) nsSNPs are smaller in size and neutral in charge (S5 Table in S2 File).

A835D/G/V, S1507T/P/A) were bigger and more hydrophobic than the wild type. T1203R/K/I have positively charged mutated residues. Both T836P/A and T1804P got small and more hydrophobic mutated residues. In R1570G/W, R1570T/L/M and R1570S, arginine is replaced by small, neutral, and more hydrophobic residues. A835D/G/V has small but more hydrophobic mutated residues (S6 Table in S2 File). Consequently, the protein's intermolecular and intramolecular interactions may be disrupted by a mutation of the residue.

### 3.9. Prediction of destabilizing effects of nsSNPs upon *TSC1* and *TSC2* gene

Destabilization analysis was subsequently done to evaluate the effects of nsSNPs on the three-dimensional structure of specific domains, further validating the structural insights provided by Project HOPE. Among 8 nsSNPs in the rhogtpase domain, 4 nsSNPs were found to destabilize by all three programs. SDM program found S237Y/F, V234E and S201P/T stabilizing, whereas only mCSM found S237C, T235I, S263C as destabilizing (Table 7). In the Gap domain, R1570G/T/L/M/S/W, S1653P and T1627S are found to be destabilizing by all three programs. S1653F, T1627N/I are found to be destabilizing by both mCSM and DUET. mCSM program didn’t recognize any nsSNPs as stabilizing. In tuberin domain, A835G/D and T836S/P are predicted to reduce stability by all three programs. S758F and T836A may cause destabilization as determined by both mCSM and DUET. Only mCSM found S758C and A835V as destabilizing mutations (Table 8).

**Table 7:** 3D structure destabilization analysis of *TSC1.*

SNP	A.A. position	mCSM	SDM	DUET
		DDG Value (kcal/mol)	DDG Value (kcal/mol)	DDG Value (kcal/mol)
rs184654520	G236E	−1.223	−1.9	−1.29
	G236A	−0.403	−1.13	−0.35
rs184667211	L161P	−1.145	−4.41	−1.832
rs213213507	S237Y	−0.594	0.15	−0.482
	S237C	−0.081	0.66	0.262
	S237F	−0.842	0.56	−0.602
rs213213560	T235S	−0.197	−0.28	0.068
	T235I	−0.118	1.40	1.065
rs213223545	L90P	−1.695	−4.41	−2.389
rs213200339	S263F	−1.335	−0.83	−1.356
	S263C	−2.345	0.65	0.85
rs213213568	V234E	−2.05	0.58	−2
rs213215407	S201P	−0.336	0.23	−0.057
	S201T	−0.647	0.6	−0.203

DDG values (kcal/mol) represent the change in Gibbs free energy upon mutation. Negative values (DDG < 0) suggest decreased protein stability, while positive values (DDG > 0) suggest increased stability

**Table 8:** 3D structure destabilization analysis of *TSC2.*

SNP	A.A. Change	mCSM	SDM	DUET
		ΔΔG (Kcal/mol)	ΔΔG (Kcal/mol)	ΔΔG (Kcal/mol)
rs45517223	S758C	−0.329	1.16	0.129
	S758F	−1.007	1.09	−0.507
rs45517365	R1570G	−1.05	0.5	−0.88
	R1570W	−0.896	−0.47	−0.671
rs45517381	S1653F	−0.732	1.54	−0.196
	S1653C	−0.453	1.45	0.071
rs397514980	R1570T	−1.799	−1.88	−2.094
	R1570L	−0.727	−0.47	−0.724
	R1570M	−1.106	−0.78	−1.321
rs397515159	S1653P	−0.11	−0.66	−0.509
	S1653T	−0.562	1.27	0.028
rs761588986	A835G	−0.795	−0.15	−0.536
	A835D	−0.603	−0.38	−0.287
	A835V	−0.223	0.01	0.106
rs1389060581	R1570S	−2.158	−2.7	−2.648
rs1555438434	T1627N	−1.362	0.08	−1.11
	T1627S	−1.533	−1.43	−1.657
	T1627I	−0.9	0.45	−0.5
rs1555438551	R1570C	−1.865	−1.26	−2.073
	R1570G	−1.908	−0.13	−1.736
rs2151354925	T836S	−0.646	−0.83	−0.539
	T836P	−0.431	−1.19	−0.546
	T836A	−0.767	0.37	−0.47

DDG values (kcal/mol) represent the change in Gibbs free energy upon mutation. Negative values (DDG < 0) suggest decreased protein stability, while positive values (DDG > 0) suggest increased stability.

### 3.10. Functional enrichment and PPI network analysis

**Fig 4:**
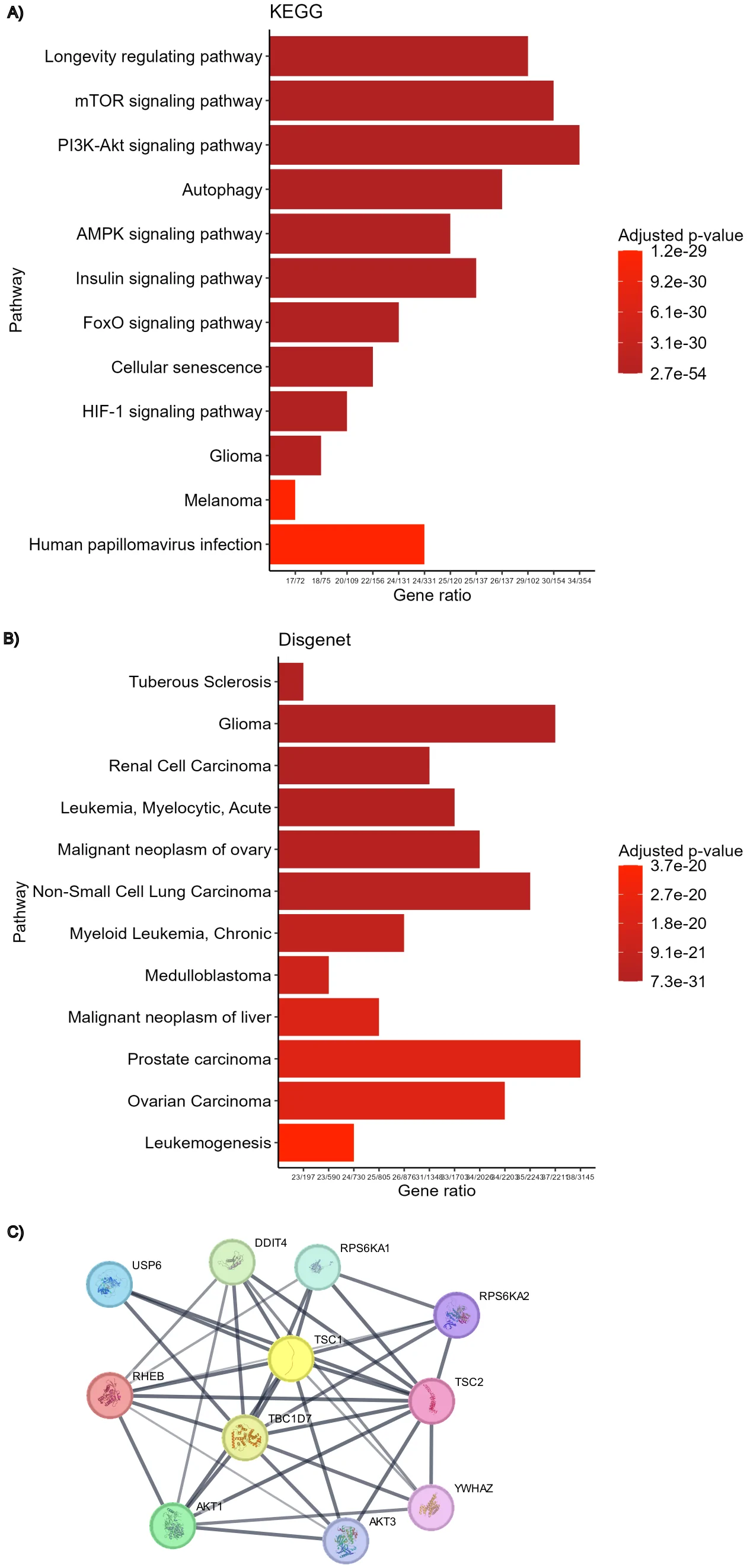
Functional enrichment and STRING analysis. A) KEGG pathways display associations of both *TSC1* and *TSC2* with different pathways. B) DisGenet shows associations with different diseases for both *TSC1* and *TSC2*.**C)** STRING server shows both *TSC1* and *TSC2* with RHEB, DDIT4, YWHAZ, RPS6KA1, RPS6KA2, AKT3, AKT1, USP6, and TBC1D7.

KEGG pathway analysis identified pathways associated with *TSC1* and *TSC2*, providing insight into how nsSNPs may disrupt these biological processes. DisGeNET analysis further revealed potential disease associations linked to altered protein function. Functional enrichment analysis was conducted with KEGG and DisGenet. KEGG pathway shows significantly enriched pathway due to *TSC1* and *TSC2* (Fig 4A). Concurrently, DisGeNET reveals significantly enriched diseases with both genes (Fig 4B). Proteins work synergistically with other proteins to influence cell signaling and other biological functions. As a result, amino acid changes in a single protein can influence other components in the interaction network. The STRING service was used to analyze the *TSC1* and *TSC2* protein interaction networks, and it identified connections with 11 interacting genes (Fig 4C).

### 3.11. Association of *TSC1* and *TSC2* gene expression with cancer survival

**Fig 5:**
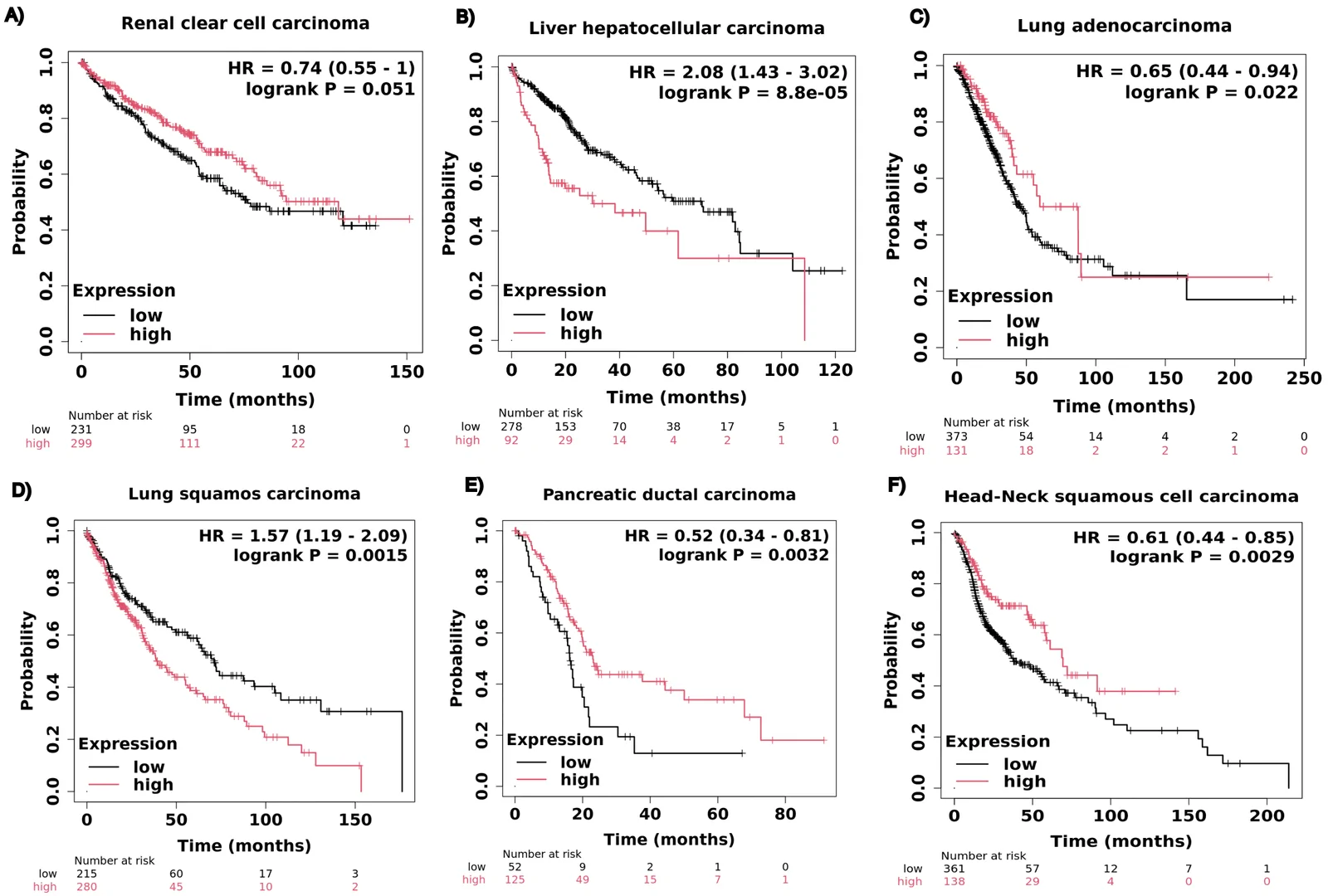
Kaplan-Meier survival plot of TSC1. A) Kidney renal clear cell carcinoma, B) Liver hepatocellular carcinoma, C) Lung adenocarcinoma, D) Lung squamous cell carcinoma, E) Pancreatic ductal adenocarcinoma. F) Head-Neck squamous cell carcinoma.

**Fig 6:**
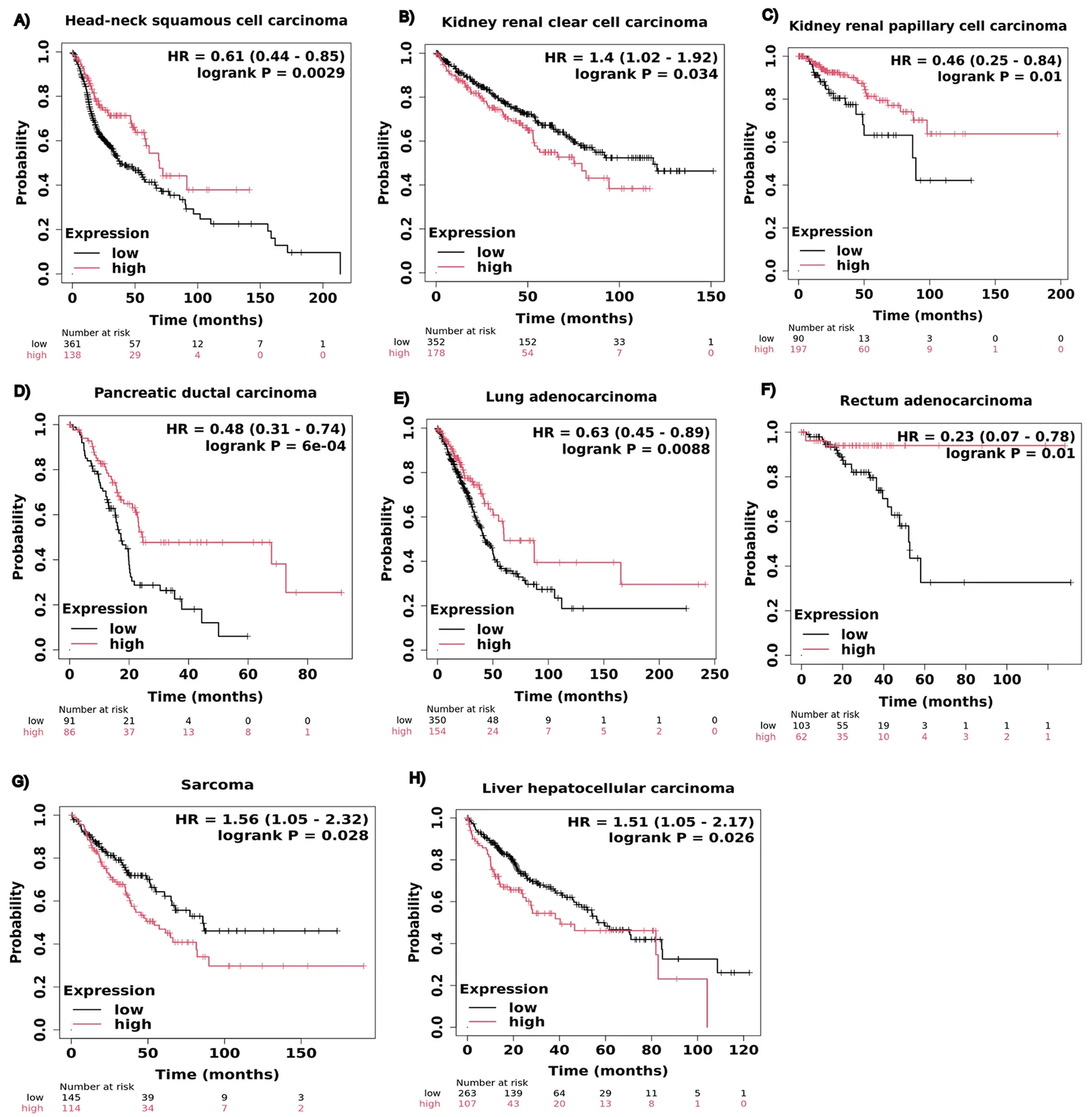
Kaplan-Meier survival plot of TSC2. A) Head and Neck squamous carcinoma, B) Kidney renal clear cell carcinoma, C) Kidney renal papillary cell carcinoma, D) Pancreatic ductal carcinoma, E) Lung adenocarcinoma, F) Rectal adenocarcinoma, G) Sarcoma, H) Liver hepatocellular carcinoma.

Kaplan-Meier survival analysis was used to determine the clinical significance of high-risk nsSNPs in TSC1 and TSC2 and their relationship to patient outcomes. Reduced TSC1 expression was linked to increased overall survival in kidney renal clear cell carcinoma, liver hepatocellular carcinoma, head and neck squamous cell carcinoma, and pancreatic ductal adenocarcinoma, whereas higher expression was associated with better results in lung cancer (Fig 5). Low TSC2 expression was associated with improved survival in lung adenocarcinoma, head and neck squamous carcinoma, kidney renal papillary cell carcinoma, and pancreatic ductal adenocarcinoma, while the opposite trend was observed in kidney renal clear cell carcinoma, liver hepatocellular carcinoma, rectal adenocarcinoma, and sarcoma (Fig 6).

### 3.12. Superimposition of wild-type protein with mutant structure

Structural comparison between wild-type and SNP-mutated *TSC1* protein using ChimeraX Matchmaker revealed 30% of residues exhibited changes in secondary structure assignment (SS fraction = 0.3), suggesting local alterations in helices, sheets, or coil formations due to SNP-induced amino acid substitutions. Superimposition of *TSC1* with SNP missense variant is present in S2 Fig and of *TSC2* in S3 Fig.

### 3.13. Molecular dynamics simulation

**Fig 7:**
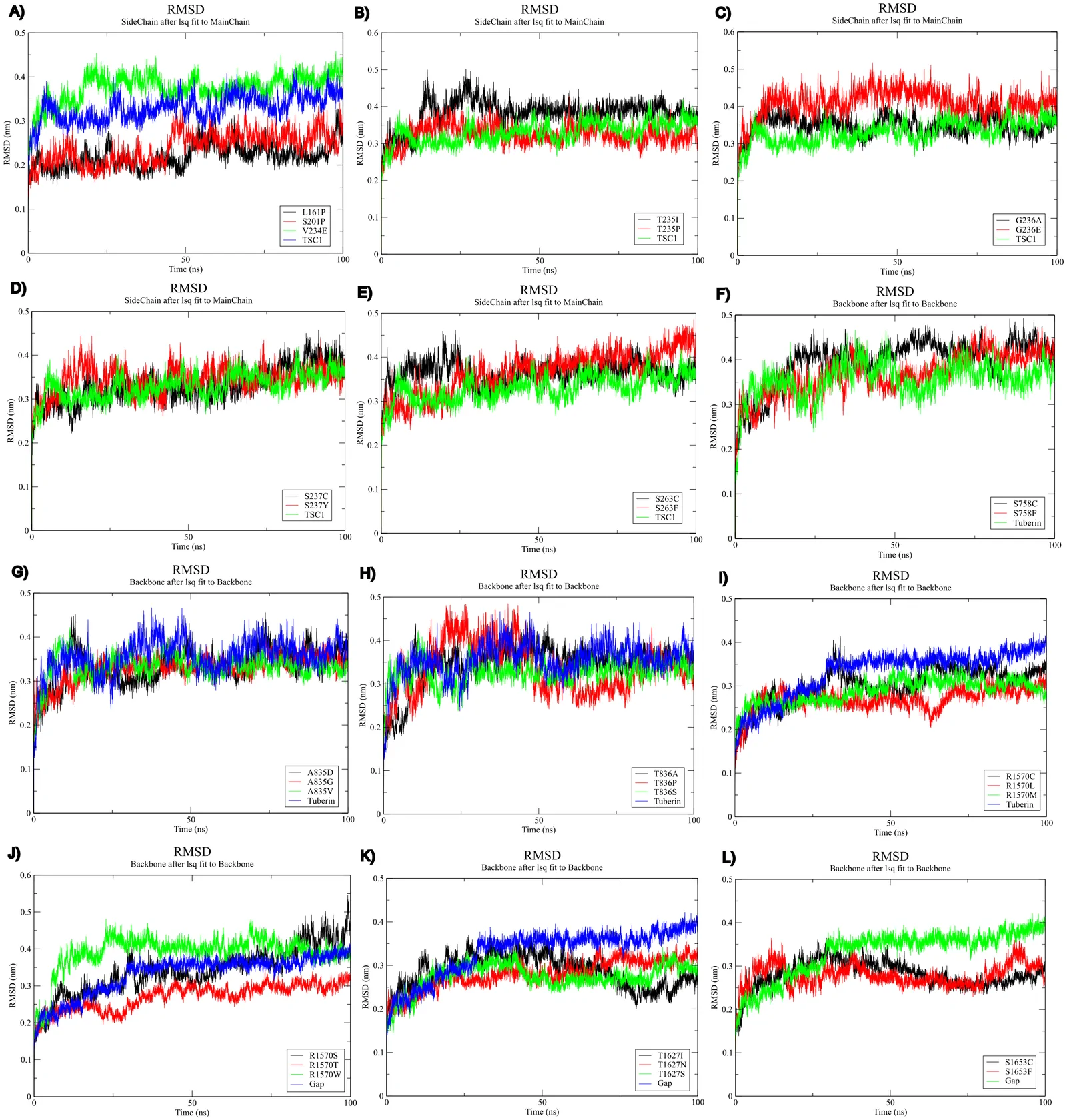
Structural stability analysis of wild-type and SNP variants of *TSC1* and *TSC2* domains. (A-E) RMSD plot of wild-type Rho-GTPase domain of *TSC1* and SNP. (F-H) RMSD plot of wild-type Tuberin domain of *TSC2* and SNP. (I-L) RMSD plot of the wild-type Gap domain of *TSC2* and SNP.

**Fig 8:**
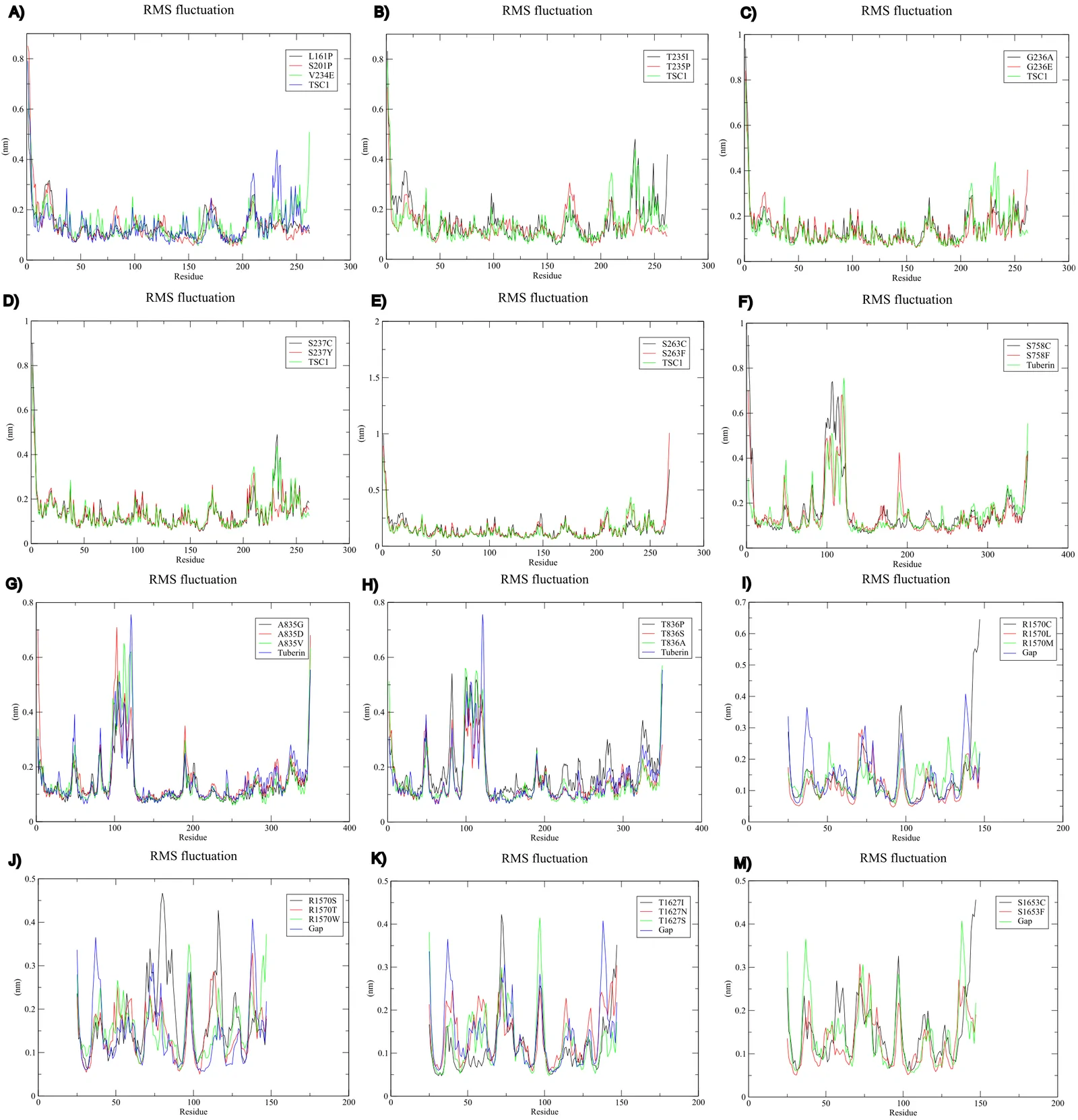
Residue-wise flexibility analysis of wild-type and SNP variants of *TSC1* and *TSC2* domains. (A-E) RMSF plot of the wild-type Rho-GTPase domain of *TSC1* and SNP. (F-H) RMSF plot of wild-type Tuberin domain of *TSC2* and SNP. (I-L) RMSF plot of the wild-type Gap domain of *TSC2* and SNP..

The RMSD value evaluates the average distance between the alpha-carbon backbones of mutant and wild-type models, and a higher RMSD value is associated with a greater deviation of the mutant structure from the wild-type. *TSC1* maintains an average RMSD of approximately 0.3–0.35 nm, showing moderate fluctuations. The V234E variant generally shows slightly higher fluctuations compared to the wild type, oscillating around 0.35–0.4 nm, and at times reaching up to 0.45 nm. The S201P variant and L161P variant exhibit similar behavior to the wild type, generally maintaining RMSD values between0.2 and 0.3 nm. The T235I variant shows slightly higher RMSD values compared to the wild-type and T235P variant. It generally fluctuates between 0.35 and 0.5 nm, indicating a potentially more dynamic or slightly less compact structure. The T235P variant demonstrates RMSD values even lower to very close to those of the wild-type TSC1, consistently fluctuating around 0.2–0.25 0.3–0.35 nm, suggesting no major structural deviation from the wild type. G236A variant shows very similar RMSD behavior to the wild type, largely overlapping the wild type. G236E variant, however, stands out with noticeably higher RMSD values between 0.35 nm and 0.5 nm, often reaching peaks close to 0.5 nm. S237C variant and S237Y variant both exhibit RMSD fluctuation between 0.3 and 0.4 nm after equilibration, that are very similar to each other and largely consistent with the wild-type protein. S263C and S263F both variant generally fluctuate around 0.35–0.4 nm, showing an upward trend towards the end of the simulation, occasionally reaching above 0.45 nm. Fig 7(A-E). RMSF plot shows the area of greater flexibility due to mutation resulting in disruptive protein folding. L161P, S201P and V234E almost matches wild type *TSC1*. T235P deviates more than the T235I variant around 150–200 residues, and T235I deviates from TSC1 around 250–300 residues. T235I deviates more than the T235P variant around 225–275 residue. Similarly, the G236E variant also shows more fluctuation in the same area than the G236A variant. Though S237C/Y variants show slight fluctuation around 200–250 residues, S263C/F show almost no deviation from the wild type Fig 8(A-E).

RMSD of the Tuberin domain is between 0.3 to 0.45. S758C variant fluctuates between 0.4 and 0.5, pointing to structural instability, but the 758F value stays between 0.3 to 0.4. The T836P variant shows a drastic RMSD value from 0.2 to 0.5, but T836A and T836S closely match wild type tuberin domain. A835G, D, and V variants remain between 0.3 to 0.4 Fig 7(F-H). RMSF of the S758C variant shows more flexibility in 100–150 residues but S758F causes flexibility in 200–225 residues. Similarly, T836P varies the most in 100–110 residues residues and slightly varies around 200–350 residues though T836A and T836S variants almost overlap wild type tuberin domain. Also, their RMSF plot mostly matches the wild-type tuberin domain Fig 8(F-H). GAP domain shows RMSD value between 0.3 to 0.4. In 1570 position, amino acid R was replaced by C, L, M, S, T, W but only R1570W and R1570S demonstrates RMSD value of 0.4 to 0.5. The others R1570C, R1570L, R1570M, R1570T remain within 0.2 to 0.35 which is less than the wild type. Both T1627I, N, S and S1653F, C variants demonstrate RMSD value of 0.2 to 0.35 Fig 7(I-L). RMSF of both R1570C and R1570W deviates from Gap domain in 100 and 150 residues though R1570S shows deviation in 75 and 120 residues. Also, R1570M deviates in 50 and 100–150 residues. S1653C fluctuates around 60 and 140 residues but S1653F doesn’t deviate much from Gap domain. Though T1627I varies from Gap domain at 75 residues but T1627S shows at 100 residues and T1627N almost matches Fig 8(I-L).

## 4. Discussion

The *TSC1* and *TSC2* genes have been extensively studied for their involvement in regulating cell growth, proliferation, and differentiation across various human tissues [57–59]. Germline mutations in *TSC1* and *TSC2* are known to disrupt the formation of the TSC protein complex, which negatively regulates mTORC1 signaling, ultimately contributing to tuberous sclerosis complex (TSC), a multisystem genetic disorder defined by tumor formation in multiple organs [60,61]. However, the *in silico* understanding of the structural and functional consequences of deleterious non-synonymous single-nucleotide polymorphisms (nsSNPs) in these genes has remained largely unexplored. The purpose of this study was to evaluate the consequences of deleterious nsSNPs in TSC1 and TSC2 at the molecular, functional, and structural levels utilizing comprehensive computational techniques. The objective was to identify the most damaging variations and prioritize them as prospective experimental validation and treatment targets. Initial screening of deleterious variants was done with multiple pathogenicity predictors of different categories to rule out bias of a single predictor, and consensus results across the tools were selected for further filtration. The selected variants were subsequently screened based on their potential to destabilize protein structure, their occurrence at evolutionarily conserved residues, and their proximity to post-translational modification sites. Variants positive across all these functions were designated as the most deleterious and further validated using MD simulations.

Twelve highly deleterious nsSNPs in the *TSC1* gene and sixteen highly deleterious nsSNPs in the *TSC2* gene were predicted to significantly compromise protein structure and function in this project. In *TSC1*, most of the nsSNPs (L161P, S201P, V234E, T235S, G236E, S237Y, S263F) are in the RhoGTPase activation domain, which helps stabilize the *TSC1*-*TSC2* complex and ensure *TSC2* can exert its GAP activity toward Rheb [62]. L90P is in the transmembrane region mediating *TSC1* binding to the cell membrane wheres S361C and I482K is in the *TSC2* binding domain, maintaining proper complex formation.These nsSNPs within the N-terminal/stability region (36–304 residues)—can reduce hamartin steady-state levels and impair *TSC1*–*TSC2*–dependent inhibition of mTOR [63,64]. In *TSC2*, S758C/F, T836P, and A835G/D/V are in the tuberin domain, which mediates interaction with *TSC1*, while R1570C, T1627S, and S1653P are in the GAP domain mediating Rheb-GAP complex formation [65]. T1023L, S1130C, T1203K, and S1207I are positioned in FOXO1 binding region. The conservation analysis provides compelling evidence for the functional importance of these residues. They all occur at highly conserved positions in case of both genes. In *TSC1*, G236E, T235I/P, and S263C/F are exposed residues, whereas L161P, S201P, V234E, and S237Y are buried within the protein’s core. In case of *TSC2*, all nsSNPs in GAP domain are exposed residues while in tuberin domain, T836P, A835G/D/V except S758C/F are buried residues. While exposed residues directly interact with the solvent and often mediate intermolecular interactions, buried residues are critical for maintaining structural integrity; therefore, these nsSNPs may significantly impair both *TSC1* and *TSC2* function. All deleterious nsSNPs of both *TSC1* and *TSC2* at phosphorylation sites indicating a high likelihood of disrupting phosphorylation. *TSC1TSC2*

Phosphorylation is a critical regulatory mechanism governing the activity, stability, and subcellular localization of the *TSC1*–*TSC2* complex. *TSC2* phosphorylation by AKT promotes mTORC1 activation whereas *TSC2* phosphorylation by AMPK inhibits mTORC1 so loss or gain of phosphorylation sites can therefore flip the regulatory outcome [66]. *TSC1TSC2TSC1TSC2* All identified deleterious SNPs were consistently classified as pathogenic. L161P followed by V234E, L90P, G236E, and L264Q showed the highest mutpred score, indicating its significant potential to affect protein structure and function. All SNPs found in *TSC1* have also been hypothesized by Mutpred to alter stability and metal binding. As divalent metal ions are essential for proper GAP-domain–mediated GTP hydrolysis [67], these alterations may directly impair regulation of Rheb-mTORC1 pathway. *TSC1TSC1TSC2*In *TSC2*, S1653P followed by S1207I, S1653F and T1804P had the highest mutpred score, indicating potential disruption of protein function. Notably, S1653F/P identified as key conformational elements of the GAP domain [68].*TSC2* that can be functionally deleterious [69]. Structural and biochemical effects of the five key nsSNPs were further explored using Project HOPE. In *TSC1*, L161P, L90P, and L264Q introduce size and conformational constraints, potentially disrupting local secondary structure, particularly α-helices. G236E/A replaces a highly flexible glycine with bulkier residues, likely restricting backbone torsion and altering local conformation. V234E and I482K/R introduce negative or positive charges, potentially disrupting native electrostatic interactions and enabling aberrant ionic contacts. Several mutations, including S237C/Y/F, S263C/F, and S201P/T, increase residue size and hydrophobicity, thus losing a hydrogen bond and cause local destabilization. cysteine substitutions (S237C, S263C) potentially form non-native disulfide bridge formation. Additionally, T235I/S and L828H are to disturb β-strand and α-helix. In *TSC2*, S758C/F(fully conserved residue), S1653C/F/T/P, S1507P/T/A, S1207N/I, and S1130C/F replace conserved serine residues with bulkier and more hydrophobic amino acids, leading to loss of hydrogen bonding and local destabilization, particularly within the tuberin and GAP domains. Mutations at T836P/A/S, T1203R/K/I, T1627N/S/I, T1804P, and T1023L/M introduce size and hydrophobicity changes, further causing steric clashes and disruption of secondary structure preferences. Notably, R1570G/W/S/T/L/M identified as 100% conserved residue change native positive charge and disrupt critical salt-bridge interactions among 1652, 1677 and 1705 residues, impairing structural rigidity within the GAP domain. Similarly, A835D/G/V alters a fully conserved residue, introducing charge (A835D) or flexibility (A835G), both detrimental to structural stability. Notably, T235S, L90P, L161P, and G236E/A are predicted to exert a destabilizing effect on the three-dimensional structure of the RhoGTPase domain by all program. R1570G/T/L/M/S/W, S1653P, T1627S in GAP domain and A835G/D and T836S/P in tuberin domain are found to cause destabilizing effect on their respective 3D structure. Both *TSC1* and *TSC2* protein plays crucial roles in several vital processes. Their interactions with RHEB, AKT1/3, RPS6KA1/2, and YWHAZ highlight direct control of mTORC1 activity through GAP function [70,71]. Association with DDIT4 underscores the role of the TSC complex in stress-induced mTOR suppression [72], while TBC1D7 stabilizes complex assembly [73]. Moreover, different carcinoma like prostate, glioma, melanoma, ovarian, renal, lung correlates to *TSC1* and *TSC2* gene in DisGenet analysis. In addition to its function in the benign tumors of TSC patients, tuberin mutations have been shown to collaborate with oncogenic mutations that facilitate the development and spread of a number of malignant cancers that affect the brain (medulloblastoma), lung, kidney (renal cell carcinomas), and breast [74–76]. TSC has also been connected to type 2 diabetes and cardiac hypertrophy by hyperactivation of AMPK and p70S6K due to tuberin dysfunction.[76]. Similar to previous findings, Kaplan-Meier plot shows both *TSC1* and *TSC2* expression is associated with overall survival in kidney renal clear cell carcinoma, pancreatic ductal adenocarcinoma. liver hepatocellular carcinoma and lung cancer. As both *TSC1* and *TSC2* don’t have a crystal structure and are very large in size, we modelled specific domains such as Rho-GTPase domain of *TSC1*, tuberin and GAP domain of *TSC2*. nsSNPs such as G236E, S263C/F and V234E with highest fluctuation indicated by both RMSD and RMSF plot, occur in the hamartin region involved in regulating cell adhesion and motility by interacting with the Rho family of GTPases. These could cause loss of adhesion to the extracellular matrix, which correlates with tumor progression in various types of human tumors and is a hallmark of transformation initiated by oncogene activation [77]. In *TSC2*, S758F and T836P showing highest fluctuation present in the tuberin region has N-terminal region, which is crucial for binding to *TSC1* [78]. MD simulation of the *TSC2* GAP domain revealed that R1570W/S exhibit elevated RMSD values, suggesting increased conformational deviation, other substitutions at the same position and variants at T1627 and S1653 displayed RMSD values comparable to or lower than the wild type, reflecting possible increased local rigidity rather than structural preservation [79]. RMSF analysis further demonstrated mutation-specific redistribution of flexibility across the domain, indicating long-range allosteric effects [80]. These findings suggest that pathogenic *TSC2* variants may impair GAP function by altering dynamic properties rather than causing complete structural unfolding [68,81]. In *TSC1*, H181P in Japanese [18], L50P in Danish [17], and L93R, N198F, and M224R are found as pathogenic missense mutations [69]. L180P, and L191H as destabilizing mutations [64], whereas L50P, L61P, L93R and V133F substitutions decreased the hydrophobicity, and R190C and R190P increased hydrophobicity of *TSC1* [63]. Also R246K, G305R, G305W, R509Q, G1035S, and R1097H amino-acid substitutions did not affect *TSC1* function [64]. In *TSC2*, R505Q, H597R, and L1624P reduce the binding affinity between *TSC1* and *TSC2* whereas S1498I, T509P, and L847P decrease signaling [20]. A recent study found Tyr194Phe, Val299Glu, Leu353Pro, Lys506Glu, Glu546Lys, Leu612Pro, Cys644Gly, Leu717Pro, Pro1497Leu, and Val1673Ala as pathogenic mutations in *TSC2* [65]. Till now, mutation reported in GAP domains are L1594M, N1643K, N1651S, P1675L, and N1681K of TSC patients. The nsSNPs identified in this project are still undocumented, so they need to be studied with special consideration to the ethnic diversity of the different populations that easily suffer from different diseases. Notably, several variants are located in close proximity to previously reported pathogenic mutations, suggesting a potential shared functional impact and reinforcing their clinical significance. These nsSNPs should be incorporated into future genetic screening panels, genotype–phenotype correlation studies, and risk stratification models. In addition, their validation in clinical cohorts would result in improved molecular diagnosis and early disease prognosis. There are also limitations to this study. While multi-tool approach containing diverse categories with a consensus strategy was adopted to reduce dependence on a single algorithm and reduce bias, it may also increase false-negative risk due to shared training biases and potentially filtering out true pathogenic variants that are underrepresented in existing databases. ClinVar variants were used as the benchmark dataset for calculating the AUC value for ROC. Several evaluated predictors, including ClinPred, REVEL, BayesDel, and AlphaMissense, were partially trained using ClinVar-derived data. Thus complete independence between the training and evaluation datasets cannot be guaranteed, which raises the possibility of circular validation. This may have contributed to the exceptionally high AUPRC values observed for these predictors and could lead to an overestimation of their true discriminative performance. Therefore, these performance metrics should not be considered representative of performance on independent external datasets. Future studies should incorporate external validation datasets that are independent of ClinVar to provide a more robust assessment of predictor performance. Computational tools often lack experimental validation and sometimes produce false positive results. The incomplete three-dimensional structure of *TSC1* and *TSC2* limits accurate assessment of the global structural impact of nsSNPs and restricts detailed visualization of their interaction within the *TSC1*–*TSC2* complex. *TSC1TSC2* Moreover, *in silico* results cannot account for tissue-specific expression, compensatory mechanisms, or interactions with other regulatory proteins and pathways. Further functional characterization, alongside a complementary clinical study, would be essential to correlate these SNPs with potential changes in protein function or disease phenotypes.

## Supporting information

S1 Fig Precision–recall curve analysis of twelve pathogenicity prediction tools. Precision is plotted against recall across different classification thresholds. The area under the precision–recall curve (AUPRC) was calculated for each tool to assess predictive performance.
(TIF)

S2 Fig 3D model structure of *TSC1* and its nsSNPs. Superimposition of wild-type *TSC1* with variant (A) L161P, (B) S201P, (C) V234E, (D)T235P, I, (E) G236E, A, (F)S237C, Y, (G) S263C, F.
(TIF)

S3 Fig 3D model structure of *TSC2* and its nsSNPs. Superimposition of wild-type *TSC2* with variant (A) S758C,F, (B) A835V,D, (C) T836P,S, (D)T1023L, M, R, (E) T1203 I, R, (F) S1207I, N, (G) S1507P,T (H) T1627I, N, S, (I) S1653P, T, (J) S1653C, F, (K) T1804P, (L) R1570W (M) R1570L, T, M, (N) R1570C, (O) R1570S.
(TIF)

S1 File S1 Table: Variant data used for calculating ROC and AUPRC of *TSC1* and *TSC2* (Excel sheet). (XLSX)

S2 File Contains Table S2 to S6. (DOCX)
