# Supplementary material for: Comprehensive analysis of non-synonymous single-nucleotide polymorphism of human TSC1 and TSC2 genes: An in silico approach
Source: PLoS One. 2026 Sep 3;21(9):e0343189. doi: 10.1371/journal.pone.0343189 (PMC13541133; doi:10.1371/journal.pone.0343189)
Supplement: Supplementary file 1 [file pone.0343189.s005.docx]

**Title: Comprehensive analysis of non-synonymous single-nucleotide polymorphism of human *TSC1* and *TSC2* genes: an *in silico* approach.**

**S1 Table: Variant data used for calculating ROC and AUPRC of TSC1 and TSC2 (Excel sheet)**

**S2 Table: Netphos 3.1 prediction of phosphorylation site.**

| Position | Residue | Phosphorylation score | Gene |
| --- | --- | --- | --- |
| TSC1 | | | |
| 162 | S | 0.853 | unsp |
| 162 | S | 0.566 | PKA |
| 235 | T | 0.531 | PKC |
| 237 | S | 0.514 | CKII |
| 263 | S | 0.729 | PKA |
| 361 | S | 0.994 | unsp |
| 361 | S | 0.652 | cdk5 |
| 361 | S | 0.547 | CKI |
| 361 | S | 0.538 | GSK3 |
| 361 | S | 0.515 | p38MAPK |
| 829 | S | 0.894 | unsp |
| 829 | S | 0.517 | cdc2 |
| TSC2 | | | |
| 758 | S | 0.98 | unsp |
| 834 | S | 0.58 | PKA |
| 834 | S | 0.58 | PKA |
| 838 | S | 0.561 | PKC |
| 838 | S | 0.543 | DNAPK |
| 1023 | T | 0.519 | CKI |
| 1203 | T | 0.738 | PKA |
| 1203 | T | 0.556 | unsp |
| 1507 | S | 0.505 | p38MAPK |
| 1507 | S | 0.505 | cdc2 |
| 1627 | T | 0.535 | PKC |
| 1653 | S | 0.995 | unsp |
| 1653 | S | 0.509 | CKII |
| 1804 | T | 0.518 | CKII |

Phosphorylation score>0.5 means high confidence in being a phosphorylation site

**S3 Table:** **MutPred2 prediction of the *TSC1* nsSNPs.**

| Mutation | Actionable/confident hypothesis | Mutpred Score | Probability | P value |
| --- | --- | --- | --- | --- |
| G236E | Gain of intrinsic disorder | 0.824 | 0.38 | 0.01 |
|  | Loss of strand |  | 0.27 | 0.02 |
|  | Altered Metal binding |  | 0.15 | 0.04 |
| G236A | Gain of Helix | 0.662 | 0.27 | 0.04 |
|  | Loss of strand |  | 0.27 | 0.03 |
|  | Altered Metal binding |  | 0.14 | 0.05 |
|  | Altered Stability |  | 0.11 | 0.04 |
| L161P | Gain of Allosteric site at R160 | 0.936 | 0.19 | 0.05 |
| I482K | Gain of B-factor | 0.782 | 0.27 | 0.01 |
| I482R | Gain of SUMOllation at K477 |  | 0.26 | 0.00072 |
|  | Altered Coiled coil |  | 0.21 | 0.02 |
|  | Gain of Ubiquitylation at I482 |  | 0.21 | 0.00053 |
|  | Gain of Methylation at I482 |  | 0.1 | 0.04 |
| S237Y | Loss of Intrinsic order | 0.78 | 0.35 | 0.04 |
|  | Gain of Allosteric site at E421 |  | 0.19 | 0.04 |
|  | Altered Metal binding |  | 0.14 | 0.04 |
|  | Gain of Sulfation at S237 |  | 0.02 | 0.02 |
| S237C | Loss of Intrinsic order | 0.688 | 0.36 | 0.04 |
|  | Altered Metal binding |  | 0.32 | 0.01 |
|  | Loss of Strand |  | 0.28 | 0.00078 |
| S237F | Loss of Intrinsic order | 0.754 | 0.36 | 0.04 |
|  | Gain of Acetylation at K238 |  | 0.2 | 0.04 |
|  | Altered Metal binding |  | 0.16 | 0.03 |
| T235I | Loss of Strand | 0.705 | 0.26 | 0.04 |
|  | Altered Metal binding |  | 0.25 | 0.04 |
| L90P | Altered Stability | 0.906 | 0.09 | 0.05 |
| L828H | Gain of Intrinsic disorder | 0.727 | 0.33 | 0.03 |
|  | Altered Coiled coil |  | 0.14 | 0.03 |
|  | Altered Stability |  | 0.13 | 0.03 |
| L264Q | Altered Metal binding | 0.883 | 0.27 | 0.02 |
|  | Altered Stability |  | 0.18 | 0.01 |
|  | Altered Transmembrane protein |  | 0.17 | 0.01 |
| S263F | Altered Metal binding | 0.773 | 0.36 | 0.00084 |
|  | Altered Transmembrane protein |  | 0.14 | 0.02 |
| S263C | Altered Metal binding | 0.757 | 0.25 | 0.03 |
|  | Altered Transmembrane protein |  | 0.14 | 0.02 |
| V234E | Gain of Intrinsic disorder | 0.918 | 0.47 | 0.00023 |
|  | Loss of Strand |  | 0.27 | 0.02 |
|  | Altered Metal binding |  | 0.25 | 0.03 |
|  | Altered Stability |  | 0.14 | 0.02 |

MutPred score >6 indicates potential pathogenicity. Probability means confidence in prediction. P-value <0.05 means significance.

**S4 Table: MutPred2 prediction of *TSC2* nsSNPs.**

| Mutation | Actionable/confident hypothesis | Mutpred score | Probability | P value |
| --- | --- | --- | --- | --- |
| S1653F | Altered Ordered interface, metal binding, Transmembrane protein | 0.885 | 0.35,0.26,0.21 | 0.00045, 0.03, 0.00039 |
|  | Loss of Relative solvent accessibility |  | 0.3 | 0.00094 |
|  | Loss of Allosteric site at Y1650, loss of sulfation |  | 0.23, 0.02 | 0.02, 0.04 |
|  | Loss of N-linked glycosylation at N1651 |  | 0.12 | 0.00098 |
| S1653C | Gain of Allosteric site at Y1650 | 0.842 | 0.25 | 0.01 |
| T1804P | Gain of Intrinsic disorder | 0.875 | 0.33, 0.25 | 0.03, 0.03 |
|  | Altered Disordered interface, Altered metal binding |  | 0.28, 0.25 | 0.04, 0.03 |
|  | Loss of Strand, Loss of Relative solvent accessibility |  | 0.26, 0.25 | 0.04 |
| T1203K | Gain of Allosteric site at W1208, Gain of N-linked glycosylation at N1205 | 0.797 | 0.29, 0.04 | 0.0027, 0.2 |
|  | Altered Transmembrane protein, Altered DNA binding |  | 0.19, 0.14 | 0.0063, 0.05 |
| T1205I | Altered Ordered interface, Transmembrane protein | 0.752 | 0.24, 0.18 | 0.04, 0.0087 |
| T1203R | Loss of N-linked glycosylation at N1205 | 0.854 | 0.04 | 0.03 |
| S1653P | Altered Metal binding, ordered interface, Transmembrane protein | 0.93 | 0.32, 0.03, 0.21 | 0.01, 0.02, 0.0036 |
|  | Loss of Relative solvent accessibility, N-linked glycosylation at N1651, Sulfation at Y1650 |  | 0.31, 0.12, 0.02 | 0.0074, 0.0098, 0.04 |
|  | Gain of Allosteric site at Y1650 |  | 0.25 | 0.01 |
| S1653T | Gain of N-linked glycosylation at N1651 |  | 0.14 | 0.0064 |
| S1207I | Altered Ordered interface, Transmembrane protein | 0.914 | 0.27, 0.17 | 0.0093, 0.0094 |
|  | Loss of Allosteric site at W1208, N-linked glycosylation at N1205 |  | 0.23, 0.04 | 0.02, 0.01 |
| S1207N | Gain of Allosteric site at W1208 | 0.877 | 0.25 | 0.01 |
| S1130F | Loss of Phosphorylation at S1130 | 0.634 | 0.57 | 0.0026 |
|  | Altered Disordered interface |  | 0.27 | 0.01 |
| T1023L | Altered Transmembrane protein | 0.872 | 0.16 | 0.01 |
| T836P | Altered Transmembrane protein | 0.806 | 0.12 | 0.03 |
| S1507P | Loss of Loop | 0.829 | 0.28 | 0.02 |
| R1570G/W | Altered Ordered interface, Transmembrane protein | 0.799,0.777 | 0.25, 0.29 | 0.03, 0.05 |
| R1570T/L/M | Altered Ordered interface, Transmembrane protein | 0.8,0.799, 0.734 | 0.25, 0.11 | 0.03, 0.04 |
| A835D | Altered Transmembrane protein | 0.81 | 0.16 | 0.04 |
| R1570S | Gain of Relative solvent accessibility | 0.767 | 0.25 | 0.03 |
|  | Altered Ordered interface, Transmembrane protein |  | 0.24, 0.1 | 0.05, 0.03 |
| R1570C | Altered Ordered interface, Transmembrane protein | 0.764 | 0.24, 0.9 | 0.04,0.05 |
|  | Loss of Relative solvent accessibility |  | 0.24 | 0.05 |

MutPred score >6 indicates potential pathogenicity. Probability means confidence in prediction. P-value <0.05 means significance.

**S5 Table: Project HOPE analysis of *TSC1***

| **SNP ID** | **A.A** | **Aminoacid properties** | **Impact on *TSC1*** |
| --- | --- | --- | --- |
| rs1846672141 | L161P | Mutant residue is smaller in size. | Might result in loss of interaction. |
| rs2132235475 | L90P | The mutant residue is smaller and both are neutral. | Proline disrupts an α-helix when not located at one of the first 3 positions of that helix; thus, the helix will be disturbed. |
| rs1846545280 | G236E/A | The mutant residue is negative, bigger and less hydrophobic. Only glycine is flexible enough to make the necessary torsion angles, mutation may disrupt local backbone into an incorrect conformation. | The wild-type residue is a glycine, the most flexible of all residues. This flexibility might be necessary for the protein's function. Mutation of this glycine can abolish this function. |
| rs2131850671 | I482K/R | A mutant residue is larger and less hydrophobic, which may lead to the loss of hydrophobic interactions at the surface or core. | The mutation is within a special region that mediates interaction with WDR45B, thus affecting the interaction. |
| rs2132135077 | S237C/Y/F | Mutated residues are bigger and more hydrophobic, which may result in loss of a hydrogen bond. | The wild-type residue is predicted to be in a turn, while the mutant residue is in another secondary structure; therefore, the local conformation will be slightly destabilized. |
| rs2132135600 | T235I/S | Mutated residue is bigger and more hydrophobic. The mutant residue is smaller, this might lead to loss of interactions. | Isoleucine causes a bump and can lead to loss of hydrogen bonds. The wild-type residue is in β-strand. Serine prefers to be in another secondary structure; thus, the local conformation will be slightly destabilized. |
| rs2131703748 | L828H | The mutated residue is bigger and more hydrophobic so it may lead to a bump. | The wild-type residue is in an α-helix, while the mutated residue does not prefer α-helices. |
| rs2132003126 | L264Q | The mutated residue is bigger and more hydrophobic so it may lead to a bump. | Hydrophobic interactions, either in the core of the protein or on the surface, will be lost due to mutation. |
| rs2132003339 | S263C/F | Alanine is a more hydrophobic residue, whereas phenylalanine is both bigger and more hydrophobic, leading to a bump. | A mutation may cause loss of hydrogen bonds. |
| rs2132135678 | V234E | Mutant residue is bigger, less hydrophobic, leading to loss of hydrophobic interaction, and negatively charged. | The wild-type residue is predicted to be located in a β-strand. The mutant residue prefers to be in another secondary structure; thus the local conformation will be slightly destabilized. |
| rs2132154057 | S201P/T | Proline is bigger and more hydrophobic. Threonine is larger. | This mutation can lead to loss of a hydrogen bond. Threonine can lead to bump. |

The difference between properties of wild-type and mutant-type amino acids regarding size, charge, and hydrophobicity is displayed.

**S6 Table: Project HOPE analysis of *TSC2***

| **SNP ID** | **A. A** | **Amino acid properties** | **Impact on *TSC2*** |
| --- | --- | --- | --- |
| rs45517223 | S758C/F | The mutation introduces a more hydrophobic residue, resulting in loss of hydrogen bonds. Wild-type residue is 100% conserved. | The wild-type residue is in its preferred secondary structure, a turn. The mutant residue prefers to be in another secondary structure; so, the local conformation will be slightly destabilized. |
| rs45517381 | S1653C | Mutation introduces a more hydrophobic residue in place of a 100%conserved wild-type residue. | The wild-type residue forms a hydrogen bond with 1651 and 1655. The mutation will cause loss of hydrogen bonds in the core of the protein and, as a result, disturb correct folding. |
|  | S1653F | The wild-type residue was buried in the core of the protein. The mutant residue is bigger and more hydrophobic. | The mutated residue is located in a domain that is important for the activity of the protein and in contact with residues in another domain. Mutation can cause an altered reaction. |
| rs372918437 | T1804P | The mutant residue is more hydrophobic than the wild-type residue. | The mutation occurs in place of a 100% conserved residue, damaging the protein. Also, it disrupts the hydrogen bond. |
| rs397515030 | T1203R/K/I | Mutant residue is positively charged, bigger, and more hydrophobic. | Hydrophobic reaction at the core or surface in tuberin domain may be lost and it also may lead to bump. |
| rs397515159 | S1653T/P | Threonine is bigger while proline is both bigger and hydrophobic. | A large and hydrophobic mutated residue disrupts interactions within the Rap-Gap domain. Proline, being a hydrophobic residue, will cause loss of hydrogen bond. |
| rs397515313 | S1207N/I | Mutant residue is bigger and more hydrophobic. | Asparagine being the more hydrophobic one, hydrophobic interactions, either in the core of the protein or on the surface, will be lost. A bigger size of the mutant may lead to a bump and hydrophobicity will cause loss of hydrogen bond. |
| rs1163066622 | S1130C/F | Mutated residue is big, hydrophobic and in place of a 100% conserved residue, | Mutation is in disordered region, and de to increased hydrophobicity, it will cause to loss of hydrogen bonds. |
| rs1555438434 | T1627N/S/I | Both Asparagine and Isoleucine are more bigger than serine and hydrophobic residue. | It may lead to loss of interaction with other molecules on surface and also affect hydrophobic interaction. Serine is smaller residue which may cause loss of external interaction. |
| rs1555510327 | T1023L/M | Mutant residue is bigger and more hydrophobic. | Bigger residue can cause bump. Also, loss of hydrogen bond is possible. |
| rs2151354925 | T836P/A/S | Alanine and serine are both small and hydrophobic. Proline is hydrophobic. | Introduction of a more hydrophobic residue in place of a conserved residue in the tuberin domain may lead to the loss of a hydrogen bond. |
| rs2151550678 | S1507P/T/A | Proline is bigger and more hydrophobic. Threonine is also bigger which can cause bump. Alanine is small and more hydrophobic residue. | Serine is in a turn whereas mutated residues are preferably in another secondary structure. Presence of bigger and more hydrophobic residue in Gap domain can cause loss of hydrogen bond thus affecting protein folding. |
| rs45517365 | R1570G/W | Mutant residue is neutral. Arginine form salt bridge among 1652, 1677 and 1705 | Glycine can disturb the required rigidity of the protein at this position. Bigger residue leads to loss of interaction with other residues in the Gap domain. |
| rs397514980 | R1570T/L/M | The mutant residue is smaller and more hydrophobic. | A mutation may lead to loss of external interaction and also loss of interaction with other molecules. |
| rs761588986 | A835D/G/V | Aspartic acid is bigger, more hydrophobic, and negatively charged. Glycine is smaller and more hydrophobic. Valine is bigger. This position is 100% conserved. | Mutation to aspartic acid may lead to loss of reaction at the core or on the surface and repulse the same charged residues. Glycine can disturb the required rigidity of the protein at this position and also cause loss of interaction. Valine may lead to a bump due to its size. |
| rs1389060581 | R1570S | The mutant residue is smaller than the wild-type residue. | This will cause a possible loss of external interactions. |

The difference between properties of wild-type and mutant-type amino acids regarding size, charge, and hydrophobicity is showed.

**S1 Fig: Precision–recall curve analysis of twelve pathogenicity prediction tools.** Precision is plotted against recall across different classification thresholds. The area under the precision–recall curve (AUPRC) was calculated for each tool to assess predictive performance.

**S2 Fig: 3D model structure of *TSC1* and its nsSNPs.** Superimposition of wild-type *TSC1* with variant (A) L161P, (B) S201P, (C) V234E, (D)T235P, I, (E) G236E, A, (F)S237C, Y, (G) S263C, F.

**S3 Fig: 3D model structure of *TSC2* and its nsSNPs.** Superimposition of wild-type *TSC2* with variant (A) S758C,F, (B) A835V,D, (C) T836P,S, (D)T1023L, M, R, (E) T1203 I, R, (F) S1207I, N, (G) S1507P,T (H) T1627I, N, S, (I) S1653P, T, (J) S1653C, F, (K) T1804P , (L) R1570W (M) R1570L, T, M, (N) R1570C, (O) R1570S.
